# Chemical Shift-Dependent Interaction Maps in Molecular Solids

**DOI:** 10.1021/jacs.3c04538

**Published:** 2023-07-13

**Authors:** Manuel Cordova, Lyndon Emsley

**Affiliations:** † Institut des Sciences et Ingénierie Chimiques, Ecole Polytechnique Fédérale de Lausanne (EPFL), CH-1015 Lausanne, Switzerland; ‡ National Centre for Computational Design and Discovery of Novel Materials MARVEL, École Polytechnique Fédérale de Lausanne (EPFL), CH-1015 Lausanne, Switzerland

## Abstract

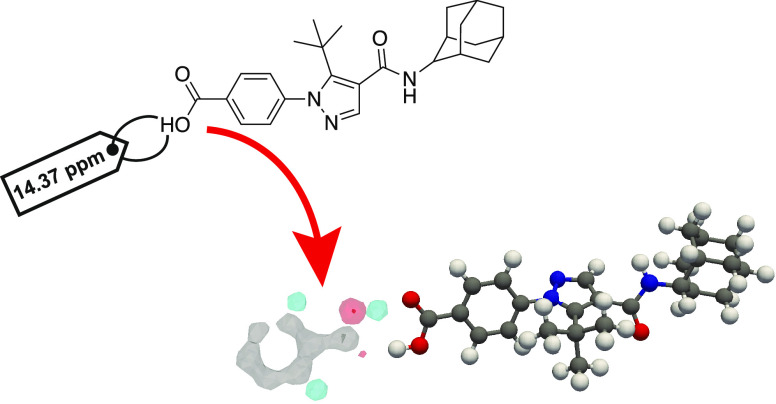

Structure determination of molecular solids through NMR crystallography relies on the generation of a comprehensive set of candidate crystal structures and on the comparison of chemical shifts computed for those candidates with experimental values. Exploring the polymorph landscape of molecular solids requires extensive computational power, which leads to a significant bottleneck in the generation of the set of candidate crystals by crystal structure prediction (CSP) protocols. Here, we use a database of crystal structures with associated chemical shifts to construct three-dimensional interaction maps in molecular crystals directly derived from a molecular structure and its associated set of experimentally measured chemical shifts. We show how the maps obtained can be used to identify structural constraints for accelerating CSP protocols and to evaluate the likelihood of candidate crystal structures without requiring DFT-level chemical shift computations.

## Introduction

Atomic-level structure determination of molecular solids is a critical step in the rationalization of their physical properties.^1,2^ This is particularly important for pharmaceutical compounds, where the three-dimensional structure determines key properties of drugs delivered in either crystalline or amorphous form, such as solubility and bioavailability.^2−4^

While single-crystal X-ray diffraction (XRD) is the gold standard for determining structures in crystalline materials, the method relies on long-range order and requires large single crystals which may be challenging to obtain.^5^ In contrast, NMR chemical shifts are local probes of local atomic environments around nuclei and thus do not require such long-range order. This has allowed the combined use of solid-state NMR, crystal structure prediction (CSP) protocols, and chemical shift computations using density function theory (DFT) for structure determination in a variety of powdered and disordered solids.^6−14^

However, crystal structure determination by NMR is still a challenging process, in part due to the large space of candidate crystal structures to explore. We have previously shown how incorporating experimental chemical shifts in CSP procedures can accelerate the generation of candidate crystal structures compatible with NMR experiments.^8^ Nonetheless, obtaining direct structural constraints could further accelerate this process.

In crystalline molecular solids, preferential interactions have previously been identified using full interaction maps (FIMs),^15^ where the propensity for interactions between pairs of functional groups are probed based on statistics extracted from the Cambridge Structural Database (CSD).^16^ This allows the identification of potential intermolecular interactions in crystalline materials, which can qualitatively inform on the intermolecular packing and be used to evaluate the relative stability of different polymorphic forms. While FIMs are useful to predict preferred noncovalent interactions in molecular solids, their use in the validation of potential crystal structures based on experimental data is limited. The construction of such maps driven by experimental properties could thus help validate potential candidates in crystal structure determination.

We have previously shown^17^ how chemical shifts in organic solids could be assigned in a probabilistic manner by comparing the measured shifts to statistical distributions of shifts obtained using ShiftML,^18,19^ a machine learning model of chemical shifts, on structures extracted from the CSD.^16^ The method involved identifying matching covalent environments in the database for each atomic site queried and obtaining the associated predicted shifts to construct the statistical distributions. One key particularity of this approach is the ability to obtain local atomic environments based on a purely covalent (2D) representation of the atomic site queried, which enabled probabilistic assignment without requiring knowledge of the three-dimensional structure of the molecule or intermolecular interactions.

Here, we construct three-dimensional atomic density maps similar to the previously reported FIMs, constructed from local atomic environments from the CSD database extracted from the previous database of local atomic environments and associated predicted chemical shifts.^17^ The atomic density maps can be considered as three-dimensional probability functions to find an atom of a given element at a given point in space in the selected environments. By selecting only environments with predicted shifts matching the experimental value, we show how the resulting chemical shift-dependent interaction maps (SIMs) predict key interactions present in the crystal structures of the samples of AZD8329 (form 1 and form 4), decitabine, and lisinopril dihydrate studied here. The SIMs obtained are compared to chemical shift-independent interaction maps (IIMs), constructed analogously from local atomic environments selected without targeting a particular chemical shift. The differences between these maps enable the identification of noncovalent interactions either promoted or reduced by applying the chemical shift constraint in the construction of the atomic density maps.

The SIMs presented here are particularly sensitive to hydrogen bonding and to the proximity of aromatic rings in the crystal packing, the latter being related to aromatic ring currents. While nucleus-independent chemical shift (NICS) maps can explain the shifts observed for nuclei in the vicinity of aromatic rings,^20−24^ the SIMs do not require the three-dimensional structure of the material to predict the presence of neighboring aromatic rings directly from experimental shifts.

## Materials and Methods

The method presented here was applied to AZD8329 (form 1 and form 4), decitabine, lisinopril dihydrate, and AZD5718. All experimental chemical shifts and crystal structures of the organic crystals studied here have been previously reported.^7,13,14,25^ The database of crystal structures and associated chemical shifts is a subset of the Cambridge Structural Database (CSD)^16^ for which chemical shift predictions were previously performed using ShiftML, a machine learning model of chemical shifts for molecular solids,^18^ in order to assign chemical shifts in a probabilistic manner.^17^ Here, we recomputed the chemical shifts using the updated model ShiftML2^19^ and extended the database to all structures available for chemical shift prediction using ShiftML2 as described in ref (17). The database now comprises over 338,000 crystal structures.

The construction of the SIM and IIM for a given covalent environment and associated shift involves identifying local atomic environments in the database that match the covalent environment, selecting 1000 environments either randomly or using the chemical shift as a constraint in the selection process to construct the IIM and SIM, respectively, aligning the selected environments on defined atoms in the covalent environment and extracting the three-dimensional atomic density maps by summing 3D Gaussians placed at each atomic position for each element found in the local atomic environments. The complete procedure is described step by step in more detail below. With the current database, the method can in principle be applied to compounds containing any subset of the 12 elements present in the database (H, C, N, O, S, F, P, Cl, Na, Ca, Mg, K).

**Figure 1:**
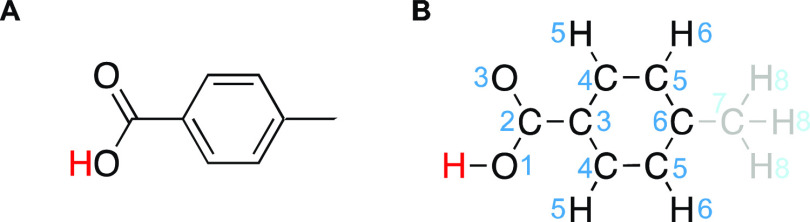
(A) Molecular structure of 4-methylbenzoic acid and (B) its associated graph representation around the carboxylic acid proton (red) up to w = 6 bonds away. Blue numbers indicate the number of bonds away from the red proton for each node (atom) in the graph representation. Atoms further than 6 bonds away are grayed out in (B).

For each ^1^H and ^13^C site, as well as bonded ^13^C-^1^H sites in each molecule, corresponding local atomic environments in the database were obtained by identifying covalent environment descriptors matching that of the atomic site. The descriptor, described previously,^17^ is a graph representing atomic species as nodes and covalent bonds as edges for all atoms within *w* bonds away from the central atomic site, as illustrated in Figure 1. A match is identified by isomorphism between the compared graphs. Importantly, this descriptor does not contain any information about the three-dimensional structure of the molecule nor intermolecular interactions, allowing for searches directly from the molecular (two-dimensional) structure, without requiring knowledge of the geometry of the molecule nor packing in the crystal structure. For each atomic site, we initially set *w* to a value of six and reduced it until the number of matches was found to be higher than 3000.

Each database instance contains the crystal structure and atomic site corresponding to the covalent environment descriptor searched for, as well as its associated ShiftML2-predicted chemical shift and predicted uncertainty. The local atomic environment corresponding to each database instance was defined as all atoms within a sphere with a radius of 7 Å centered at the atomic site. When required, the unit cell of crystal structures from the database was repeated in order to completely fill the defined sphere with atoms from the selected structures.

The local atomic environments corresponding to each atomic site were then aligned to a chosen conformation of the molecule under study by minimizing the root-mean-square displacement (RMSD) between the positions of selected atoms in the environments through rotation and translation of the whole environments. Although we aligned all environments to the molecular conformation found in the experimental crystal structure of each compound, we note that this alignment can be performed on any conformation without loss of generality, provided that the geometry of the atoms selected for the alignment does not change upon conformational changes. To ensure that, we aligned between three and four atoms, all within at most two bonds of each other, except for rigid molecular motifs such as phenyl rings and carboxylic acids, where we allowed more distant atoms to be aligned. The set of atoms selected for alignment around each atomic site is described in Supplementary Tables S1–S12.

For each atomic site, we randomly selected 1000 environments to obtain the average environment around the selected atomic site regardless of its chemical shift, and then another 1000 environments were selected by drawing numbers from a Gaussian distribution centered at the experimental chemical shift and with a width given by the expected uncertainty of the ShiftML2 prediction,^19^ which corresponds to 0.5 ppm for ^1^H and 5 ppm for ^13^C. For each number drawn, the environment with the closest chemical shift was selected. The environments for bonded ^13^C-^1^H sites were selected similarly by drawing numbers from a two-dimensional Gaussian distribution centered at the experimental ^13^C (δ_13C_^exp^) and ^1^H (δ_1H_^exp^) shifts and with a width of σ_13C_ = 5 and σ_1H_ = 0.5 ppm in the first (^13^C) and second (^1^H) dimensions, respectively. The environment with the closest correlated chemical shift was identified by defining the distance *d* from the experimental chemical shift as
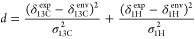
1where δ_13C_^env^ and δ_1H_^env^ are the ^13^C and ^1^H chemical shifts of the bonded pair of atoms in the environment, respectively.

Three-dimensional atomic density maps were generated by summing three-dimensional Gaussian functions with a width σ = 0.5 Å placed at the atomic positions *r⃗*_*a*_*i*__ of the aligned local environments
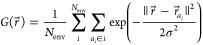
2Individual atomic density maps were constructed for each element present in the set of selected environments. The Gaussian functions where not normalized, and this leads to a value of 1 at a given position if an atom of a given element is found at that position in all selected environments. Each atomic density map was evaluated on a 31 × 31 × 31 cubic grid centered at the atomic site and with 12 Å sides. This corresponds to a spatial sampling of 0.4 Å. The size of the grid was chosen to be close to the 7 Å radius sphere used to construct the descriptor to perform chemical shift predictions using ShiftML2.^19^ The atomic density maps obtained using randomly selected environment represent chemical shift-independent interaction maps (IIMs), and those obtained from environments selected around the measured chemical shifts represent chemical shift-dependent interaction maps (SIMs). All IIMs and SIMs constructed here are shown in Figures S2–S16.

The score *s*_*i*_ of a local atomic environment *i* in a candidate crystal representing its compatibility with the measured chemical shift was evaluated as the overlap between the atomic density map of that local environment *G*_*i*_^cand^(*r⃗*) and the difference between the corresponding SIM (*G*_*i*_^SIM^(*r⃗*)) and IIM (*G*_i_^IIM^(*r⃗*))
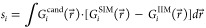
3This score thus represents how much the local atomic environment is promoted by the SIM compared to the IIM. In practice, we set values in the difference between SIM and IIM at a given point with a magnitude below 0.01 to zero in order to mitigate noise in the difference maps. Here, a positive value of *s*_*i*_ indicates that the corresponding atomic environment is more compatible with the SIM than with the IIM. A value of zero indicates that the candidate is equally promoted by the SIM and the IIM. If the atomic environment is more compatible with the IIM than with the SIM, then a negative value will be obtained. The global score for a candidate crystal was computed as the mean of all considered local atomic environment scores. Here, we discarded the maps that correspond to ambiguous assignments (e.g., aromatic rings and CH_2_ groups) from the computation of global scores in order to avoid ambiguities in the scores. Ambiguity arises in such groups due to the mapping of the 2D descriptors to atomic sites in the chosen 3D conformation. It is not possible to determine *a priori* the assignment of, e.g., the two different protons in a CH_2_ group yielding two different chemical shifts without knowledge of the crystal structure.

When comparing sets of candidate structures, we normalized the scores obtained by subtracting the mean score across all candidates from the global score obtained for each candidate. This removes any systematic tendency observed within the set of candidates, leaving only variations between candidates. The final normalized scores obtained thus indicate, within the set of candidate structures considered, which candidates are better matching the SIMs than the IIMs, corresponding to a positive score. While these scores may not be able to definitively identify the correct candidate crystal, they can allow the preselection of potential crystal structures by discarding structures displaying strongly negative scores.

## Results and Discussion

The method presented here was applied to AZD8329 (forms 1 and 4), decitabine, lisinopril dihydrate, and AZD5718, using the previously reported experimental ^1^H and ^13^C chemical shifts of these compounds.^7,13,14,25^

For each atomic site considered in each compound, the database was first queried to obtain the local atomic environments matching the covalent environment queried, as well as their associated chemical shift. The IIMs and SIMs were subsequently constructed by selecting 1,000 environments either randomly or with associated shifts close to the experimental value, respectively, as described in the Methods section. The whole process can be performed directly from the chemical structure of the molecule studied and the set of assigned chemical shifts and can thus be performed, e.g., in parallel to the construction of CSP candidates. In general, obtaining each interaction map takes under an hour on a single CPU core and can be straightforwardly parallelized. Once the interaction maps are constructed, computing scores for candidate crystal structures typically takes up to a few seconds per structure, against hours to days of CPU time to obtain chemical shifts using DFT, and scales linearly with the number of atoms in the structure (against a cubic dependence for GIPAW DFT). The method presented here thus provides great potential to facilitate structure determination by NMR.

**Figure 2:**
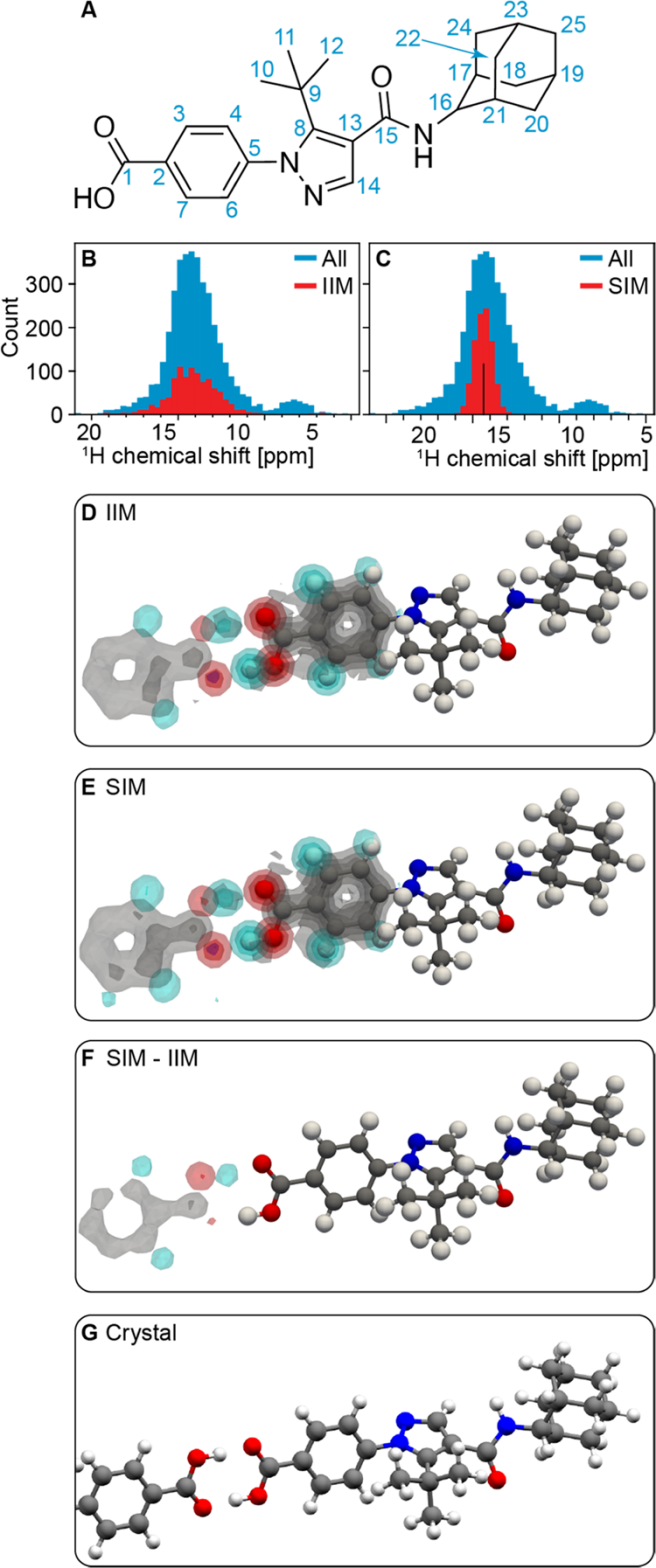
(A) Labeling scheme of AZD8329. (B, C) Histogram of chemical shifts associated with structures from the database matching the covalent environment of proton labeled 1 (blue) and of the 1,000 structures (red) selected either randomly to construct the IIM (B) or sampled around the experimental chemical shift (vertical black line) measured in AZD8329 form 1 to construct the SIM (C). (D, E) Three-dimensional contour levels of the IIM and SIM of proton 1 in AZD8329 obtained using eq 2 from the structures selected in (B) and (C), respectively. Contour levels are drawn at values of 0.2, 0.4, 0.6, and 0.8. (F) Three-dimensional contour levels of the difference of atomic density between the SIM and IIM. Contour levels are drawn at values of 0.05, 0.1, 0.15, and 0.2. (G) Intermolecular hydrogen bonding motif of the proton labeled 1 in the crystal structure of AZD8329 form 1.

Figure 2 shows the atomic density maps obtained for the carboxylic acid proton of AZD8329 form 1. By aligning 1,000 environments randomly selected regardless of the chemical shifts (Figure 2B) or such that their predicted chemical shift is the same as the experimental value, to within the prediction error (Figure 2C), we obtain the atomic density maps shown in Figure 2D,E. Both maps were found to be similar and to predict a carboxylic acid dimer in at least 20% of the environments aligned. By displaying the difference between the maps obtained with and without the experimental chemical shift of the carboxylic acid proton (Figure 2F), the dimer was found to be promoted in the ensemble of local atomic environments that match the experimental chemical shift, by at least 5% of the total number of environments aligned. As shown in Figure 2G, the dimer is indeed present in the crystal structure of AZD8329 form 1, which is consistent with the higher atomic densities found at the positions of the atoms in the dimer in the environments selected around the experimental chemical shift compared to the environments selected regardless of the shift.

As mentioned in the Methods section, the maps were aligned to the conformer found in the crystal structure but can be generated around any conformation, allowing the visualization of preferred interactions without any prior knowledge of the crystal structure of the compound studied.

**Figure 3:**
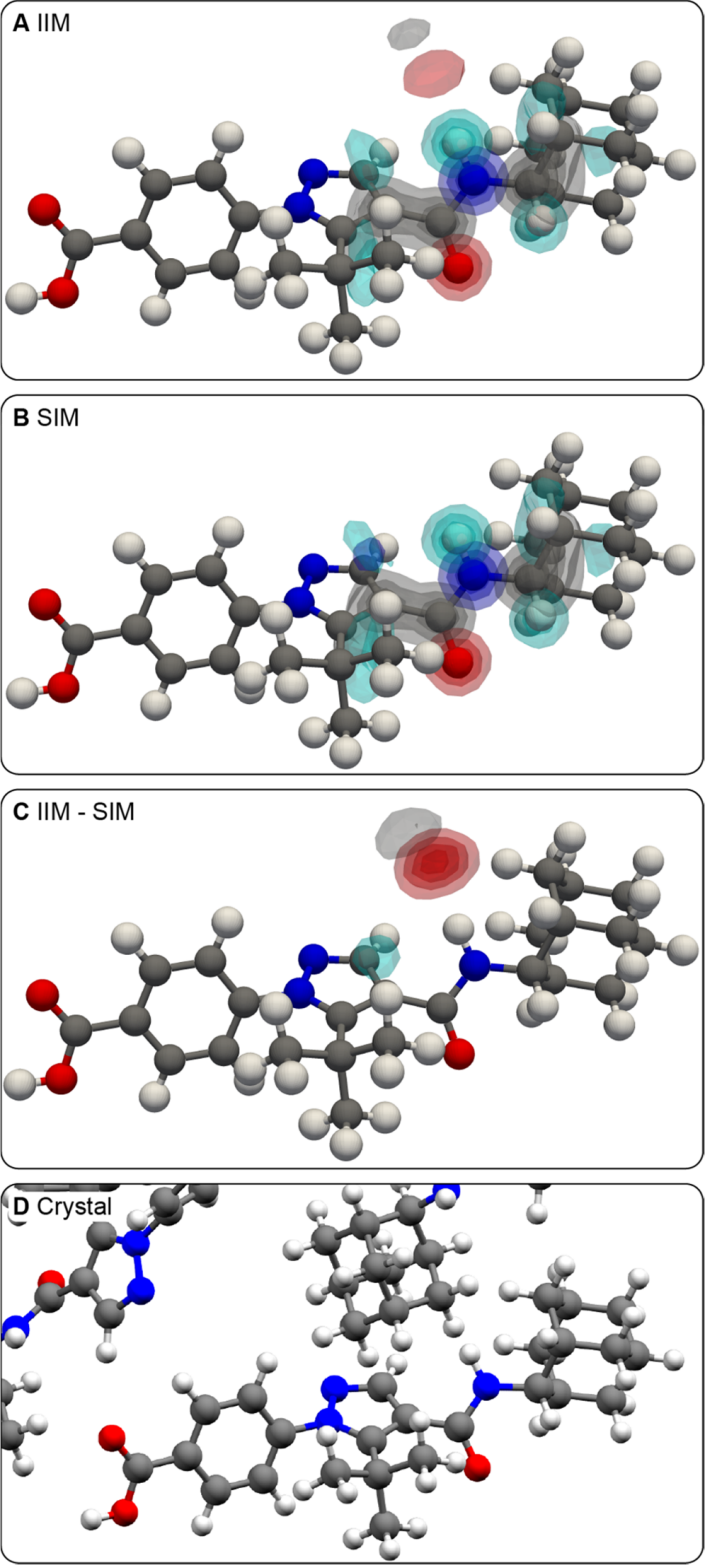
(A, B) Three-dimensional contour levels of the IIM and SIM of the NH proton of AZD8329 form 1, respectively. Contour levels are drawn at values of 0.2, 0.4, 0.6, and 0.8. (C) Three-dimensional contour levels of the difference of atomic density between the IIM and SIM. Contour levels are drawn at values of 0.05, 0.1, 0.15, and 0.2. (D) Local atomic environment of the NH proton in the crystal structure of AZD8329 form 1.

**Figure 4:**
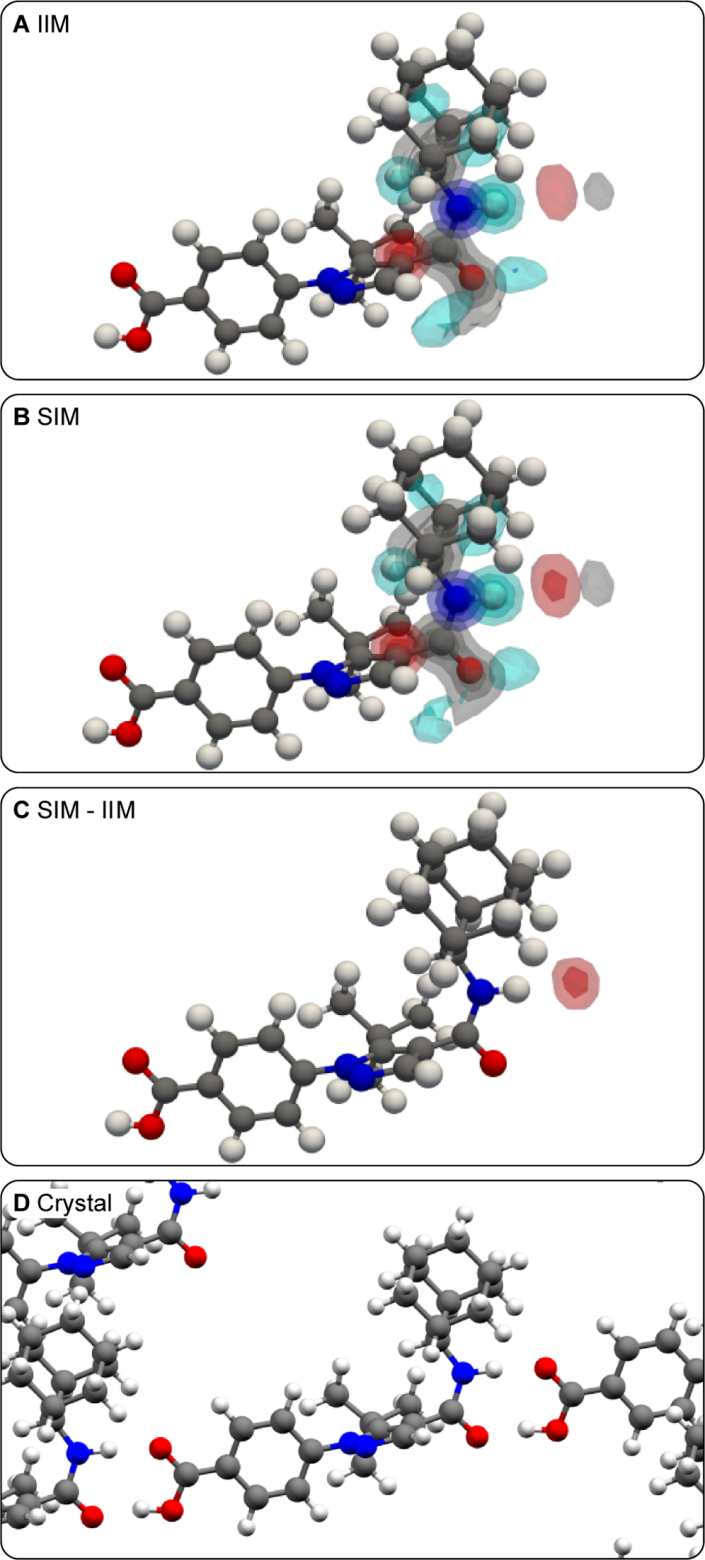
(A, B) Three-dimensional contour levels of the IIM and SIM of the NH proton of AZD8329 form 4, respectively. Contour levels are drawn at values of 0.2, 0.4, 0.6, and 0.8. (C) Three-dimensional contour levels of the difference of atomic density between the SIM and IIM. Contour levels are drawn at values of 0.05, 0.1, 0.15, and 0.2. (D) Local atomic environment of the NH proton in the crystal structure of AZD8329 form 4.

Figures 3 and 4 show the atomic density maps obtained around the NH proton in AZD8329 forms 1 and 4, respectively. In form 1 (Figure 3A,B), the selection of local environments with associated chemical shifts around the experimental value (see Figure S17) was found to reduce the atomic density of oxygen in contact with the NH proton. The reduction in atomic density corresponds to a difference of at least 20% of the local atomic environments aligned, as seen in the difference map shown in Figure 3C. We note that Figure 3C shows the difference between atomic densities obtained from randomly selected environments and those selected around the experimental chemical shift (IIM–SIM), unlike those shown in Figure 2F and below. This allows us to identify interactions that are less likely than on average when considering the experimental chemical shift. Indeed, the NH proton is not hydrogen-bonded in the crystal structure of AZD8329 form 1 (Figure 3D).

In AZD8329 form 4, the atomic density maps obtained for local atomic environments around the same NH proton with associated chemical shifts close to the experimental value (see Figure S17) were found to promote hydrogen bonding to oxygen atoms (Figure 4A–C). This is in agreement with the hydrogen bond found in the crystal structure of form 4 (Figure 4D).

In the case of form 4, we also note that in this case, the maps in Figure 4A,B do not capture the cis conformation of the amide group found in the crystal structure. This suggests that the overwhelming majority of amides in the database display a trans conformation and/or that the conformation is not captured in the chemical shift of the NH proton. We note that none of the ^1^H or ^13^C shifts considered was able to capture the cis conformation.

The atomic density maps obtained around the carboxylic acid proton in AZD8329 form 4, shown in Figure S2, were found to promote hydrogen bonding of the proton, which is consistent with the crystal structure of the material. However, the difference map was found to promote the carboxylic acid dimer found in the structure of form 1, and which is not present in form 4. This can be explained by bias in the database, where most hydrogen-bonded carboxylic acid groups are dimers. Experimental validation of the presence of a carboxylic acid dimer can be obtained using complementary methods such as, e.g., a BABA-xy16 experiment.^26,27^ The CH protons, as well as carbon environments obtained were not found to promote any significant interaction or conformation in the material. The superposition of interaction maps generated around all ^1^H, ^13^C, and ^1^H-^13^C sites are provided for AZD8329 form 1 and 4 in Figure S2–S7.

**Figure 5:**
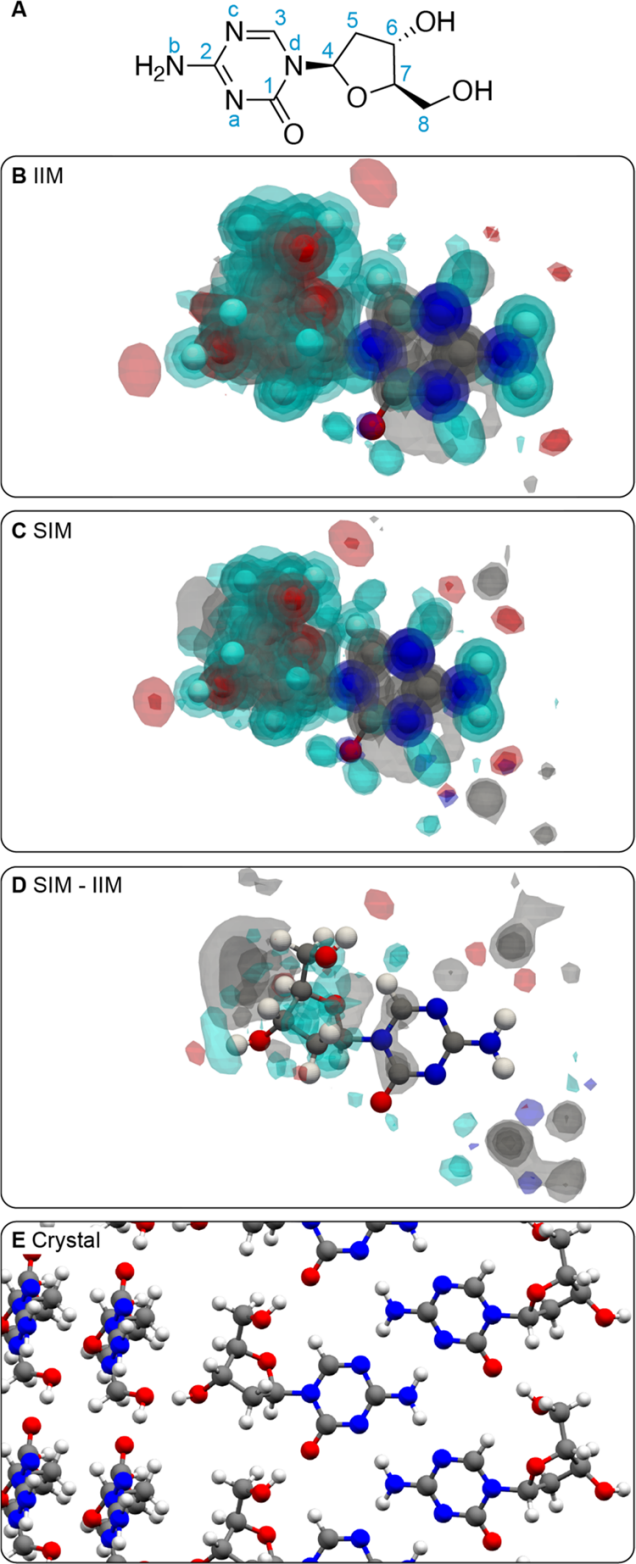
(A) Labeling scheme of decitabine. (B, C) Superposition of three-dimensional contour levels of the IIMs and SIMs of all protons in decitabine, respectively. Contour levels are drawn at values of 0.2, 0.4, 0.6, and 0.8. (D) Three-dimensional contour levels of the difference of atomic density between the SIMs and IIMs of each proton in the molecule. Contour levels are drawn at values of 0.05, 0.1, 0.15, and 0.2. (E) Local environment around a decitabine molecule in the crystal structure.

Figure 5 shows the atomic density maps generated around all protons in decitabine. Both density maps constructed from randomly selected environments and environments with associated shifts close to experimental values display hydrogen bonding of both protons in the amine, both OH protons, nitrogen labeled **a** and **c** in Figure 5A, and the oxygen labeled **1** in at least 20% of the environments used to construct the atomic density maps (Figure 5B,C). The difference map shown in Figure 5D shows that the experimental ^1^H chemical shifts are associated with a higher degree of all hydrogen bonding identified above than on average by at least 5% of all environments aligned. This is confirmed in the crystal structure, where all the aforementioned atomic sites are hydrogen-bonded. We note that one of the NH_2_ protons is expected to be H-bonded to a carboxylic acid moiety in the atomic density map, while it is H-bonded to a nitrogen in the crystal structure.

Figure 5D illustrates the limitations of the method presented here. First, the atomic density maps generated do not explicitly identify functional groups. For example, the hydrogen bonding partners of the OH groups in decitabine are not identified in Figure 5D, which only provides the information that the OH groups are likely to be H-bonded. Nonetheless, the shape of the atomic density maps can be used to infer the bonding partner. In addition, the method presented here is not able to disambiguate intra- or intermolecular interactions. A careful analysis of the flexibility of the molecule can however often establish the possibility of intramolecular interactions. Another limitation of the method is the identification of the hydrogen bonding acceptors around H-bonded protons. In the case of decitabine here, one of the NH_2_ protons is expected to be bonded to a carboxylic acid, although no such functional group is present in the crystal structure. This artifact is due to bias in the database used to construct the atomic density maps, where in this case, most environments that match the observed chemical shift display hydrogen bonding interactions with carboxylic acid groups. However, in the absence of such a chemical group in the crystal structure, the most similar group is the aminopyrimidine-like moiety in the molecule, which is the hydrogen bonding partner observed in the crystal structure (Figure 5E). This interaction could be probed with complementary experiments such as, e.g., a ^14^N-^1^H d-HMQC experiment.^28^

In Figure 5B–D, the proton density found around the C=O group is an artifact in the maps constructed for the NH_2_ protons, which predict an NH_2_ group instead of the oxygen next to the carbon labeled 1. This is due to bias in the database.

The superposition of interaction maps generated around all ^13^C and ^1^H-^13^C sites are provided for decitabine in Figures S9 and S10.

**Figure 6:**
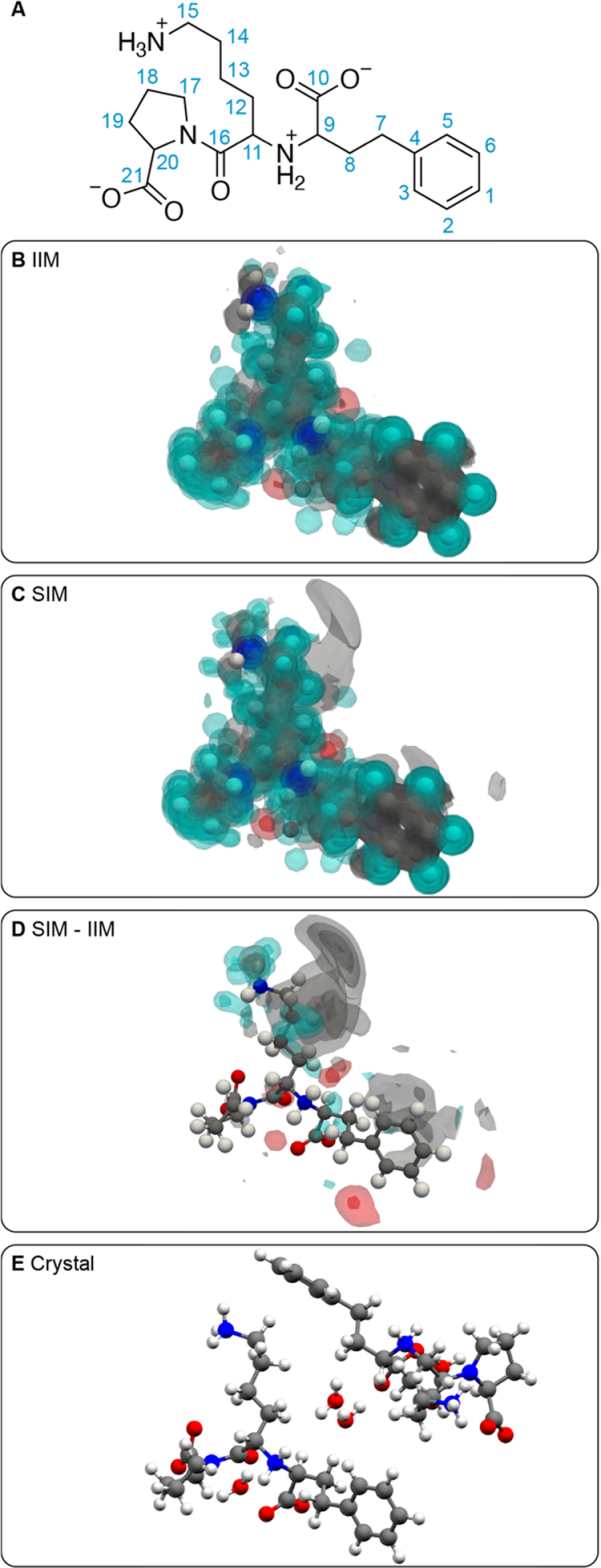
(A) Labeling scheme of lisinopril dihydrate. (B, C) Superposition of three-dimensional contour levels of the IIMs and SIMs of all protons in lisinopril, respectively. Contour levels are drawn at values of 0.2, 0.4, 0.6, and 0.8. (D) Three-dimensional contour levels of the difference of atomic density between the SIMs and IIMs of each proton in the molecule. Contour levels are drawn at values of 0.05, 0.1, 0.15, and 0.2. (E) Local environment around a lisinopril molecule in the crystal structure.

Figure 6 shows the atomic density maps generated around all protons in lisinopril dihydrate (excluding water protons since their chemical shift was not reported). The map generated around the CH_2_ protons labeled **15** using environments selected to have close chemical shifts (Figure 6C) displays a clear presence of carbon atomic density close to the protons, which is absent in the map generated using random local atomic environments (Figure 6B). This is confirmed in the difference map (Figure 6D) and corresponds to the presence of the phenyl ring of a neighboring lisinopril molecule. The unusually low shift of one of the CH_2_ protons (see Table S7 and Figure S18) is associated with the presence of an aromatic ring in its vicinity, whose ring currents induce an increased shielding of the proton. This effect has previously been extensively studied in the context of nucleus-independent chemical shift (NICS).^6,20−24^ The superposition of interaction maps generated around all ^13^C and ^1^H-^13^C sites are provided for lisinopril dihydrate in Figures S12 and S13.

**Figure 7:**
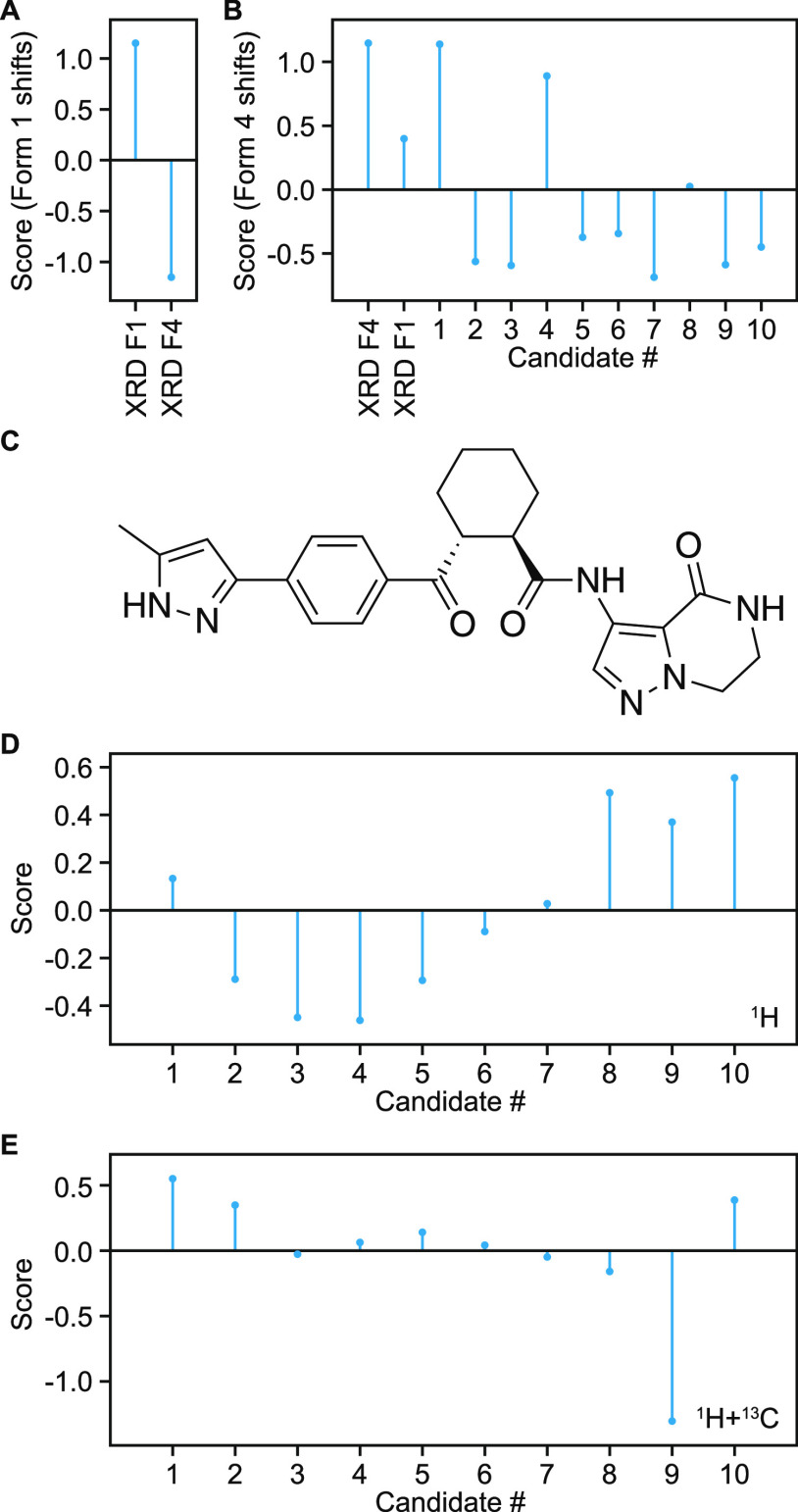
Scores obtained as described in eq 3 and averaged over all atomic environments considered for the X-ray structures of AZD8329 forms 1 and 4 using SIMs constructed using the experimentally obtained chemical shifts of (A) AZD8329 form 1 and (B) form 4. In (B), the scores obtained for a CSP set of 10 candidate structures of AZD8329 form 4 are also shown. Chemical structure of AZD5718 (C) and scores obtained for candidate structures of AZD5718 using SIMs constructed using the experimentally obtained (D) ^1^H and (E) ^1^H and ^13^C chemical shifts.

The atomic density maps presented here can be used to qualitatively evaluate the likelihood of candidate structures in chemical shift-based structure determination or can serve as the basis for the derivation of structural constraints in CSP protocols. In addition, we introduce a quantitative measure of the likelihood of candidate crystal structures based on the atomic density maps generated (see the Methods section). Figure 7A,B shows the scores obtained for the X-ray structures of forms 1 and 4 of AZD8329 when evaluated using the maps generated from the experimental ^1^H chemical shifts of all unambiguously assigned protons (see Figure S19). In addition, the evaluation of a set of 10 candidate structures is shown for AZD8329 form 4. The SIMs constructed from the experimental shifts of form 1 correctly lead to a higher score for the X-ray structure of form 1 compared to form 4 (Figure 7A). In addition, using SIMs derived from the experimental shifts of AZD8329 form 4 led to the correct identification of the X-ray structure of form 4 and candidate #1 in the CSP set to have the highest scores compared to the X-ray structure of form 1 and the other CSP candidates (Figure 7B). This indicates that the method is able to identify the correct polymorphic form of AZD8329 based on experimental chemical shifts only and highlights the ability of SIMs to identify the correct crystal structure among a set of candidates directly from the experimentally measured chemical shifts, without the need to perform any chemical shift computation for any candidate in the set. Using ^13^C or both ^1^H and ^13^C chemical shifts from AZD8329 form 1 similarly leads to a higher score for the X-ray structure form 1 compared to form 4; however, using ^13^C or both ^1^H and ^13^C chemical shifts from AZD8329 form 4 did not attribute the highest score to candidate #1 (see Figure S19).

Figure 7C–E shows the scores obtained for a CSP set of candidate structures of AZD5718 using unambiguously assigned experimental ^1^H (Figure 7D) and ^1^H and ^13^C (Figure 7E) chemical shifts. In this case, using protons only did not identify candidate #1 (i.e., the correct candidate) as having the highest score. However, adding ^13^C chemical shifts led to the correct identification of candidate #1 as best matching (see Figure S19). The superposition of interaction maps generated around all ^1^H, ^13^C, and ^1^H-^13^C sites are provided for AZD5718 in Figure S14–S16. Not unexpectedly, the scores display a weaker discriminating power compared to DFT chemical shift computation of the candidate structures and comparison to experiments^7,13^ so far, and further work will focus on improving the robustness of candidate scoring.

We note that here all CSP candidate structures of AZD8329 form 4 were originally selected by Baias et al.^7^ within 30 kJ/mol in total energy from the most stable predicted crystal structure with the cis conformation of the amide group and ordered by increasing energy. While the lowest energy candidate corresponds to the X-ray structure of AZD8329 form 4, it lies well above the lowest energy candidate generated with a trans conformation of the amide group. For AZD5718, the 10 candidate crystal structures were previously selected within 6 kJ/mol from the lowest energy candidate generated^13^ and are ordered by increasing energy. In general, there is no guarantee that the lowest energy candidate corresponds to the observed structure, and this is evident for polymorphic compounds that display several observed structures with different energies. The IIMs and the SIMs generated here do not incorporate any information or bias related to predicted energies.

## Conclusions

Here, we have presented a method to obtain three-dimensional atomic density maps of local atomic environments based on the experimental chemical shift associated to the covalent environment queried. The maps constructed can be used to visualize preferred noncovalent interactions in molecular solids directly from any random conformation of the compound studied, without requiring any prior knowledge about the conformation of molecular packing in the solid state. This can be used to qualitatively evaluate the likelihood of candidate crystal structures in chemical shift-based structure determination or to derive experimentally derived structural constraints in CSP protocols. It can also be used to generate structural hypothesis that can guide further experimental validations. We have also introduced a scoring system able to quantitatively evaluate candidate crystal structures based on experimental chemical shifts, which was found able to identify the correct candidate.

While we believe that the method presented here presents great potential to facilitate the structure determination of molecular solids by NMR, we expect it to become more powerful in the future, using larger and more diverse databases of structures with more accurate chemical shifts associated. Using larger and more diverse databases would also allow the use of the method for a broader range of compounds. Finally, we expect that managing bias in the database (e.g., the over-representation of particular functional groups) would allow the construction of more accurate SIMs.

The approach presented here is not limited to crystalline compounds, and can be used straightforwardly to identify preferred noncovalent interactions in disordered materials by using experimental chemical shifts from such disordered samples and adapting the width of the shift distributions to match the observed lineshapes, potentially made more accurate by using a database comprising distorted structures.

## Supporting Information Available

Experimental chemical shifts, aligned atoms, and supplementary data (PDF)
