# Supplementary material for: Chemical Shift-Dependent Interaction Maps in Molecular Solids
Source: J Am Chem Soc. 2023 Jul 13;145(29):16109–17. doi: 10.1021/jacs.3c04538 (PMC10375520; doi:10.1021/jacs.3c04538)
Supplement: Supplementary file 1 — ja3c04538_si_001.pdf [file ja3c04538_si_001.pdf]

Chemical Shift-Dependent Interaction Maps in Molecular Solids

Manuel Cordova[1,2], Lyndon Emsley[1,2*]

[1]Institut des Sciences et Ingénierie Chimiques, Ecole Polytechnique Fédérale de Lausanne (EPFL), CH-1015 Lausanne, Switzerland

[2]National Centre for Computational Design and Discovery of Novel Materials MARVEL, École Polytechnique Fédérale de Lausanne (EPFL), CH-1015 Lausanne, Switzerland

*Lyndon Emsley: lyndon.emsley@epfl.ch

Raw data statement. All data and code used are available from https://doi.org/10.24435/materialscloud:98-sx under the license CC-BY-4.0 (Creative Commons Attribution-ShareAlike 4.0 International).

Experimental Details

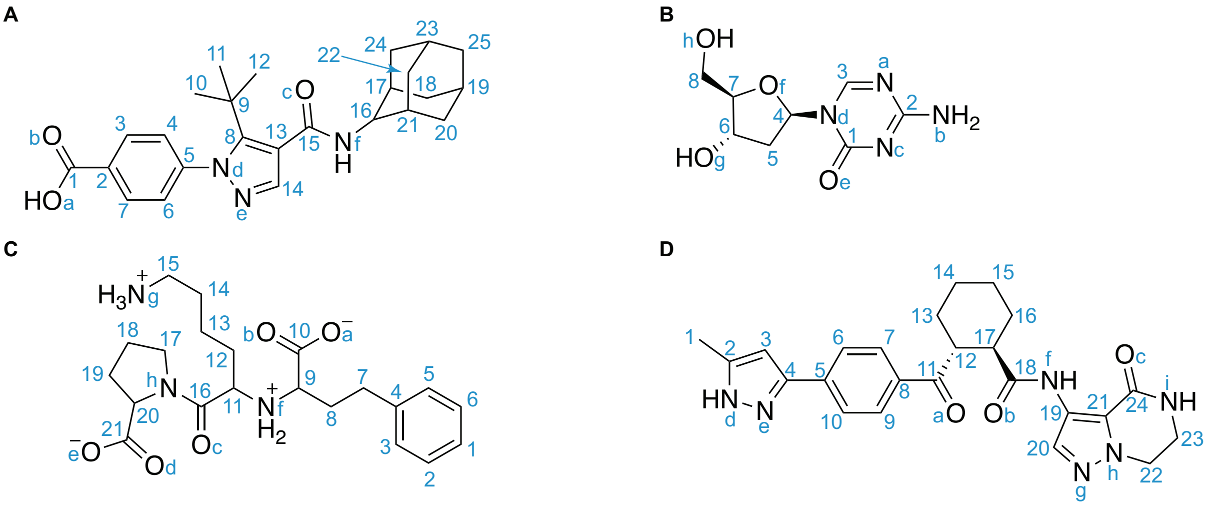

Figure S1. Labelling scheme of (**A**) AZD8329, (**B**) decitabine, (**C**) lisinopril dihydrate and (**D**) AZD5718.

Table S1. Experimental ^{1}H chemical shifts and atoms aligned for AZD8329. The chemical shifts are obtained from Ref. 1.

Label	Experimental shift, Form 1 / Form 4 [ppm]	Atoms aligned
H1	14.37 / 15.37	H^{a}, O^{a}, O^{b}, C^{1}
H3	8.46 / 9.01	H^{3}, C^{3}, O^{a}, O^{b}
H4	7.08 / 8.47	H^{4}, C^{4}, N^{d}, C^{1}
H6	8.46 / 6.92	H^{6}, C^{6}, N^{d}, C^{1}
H7	8.46 / 8.69	H^{7}, C^{7}, O^{a}, O^{b}
H10	1.01 / 0.73	C^{10}, C^{9}, C^{8}
H11	1.01 / 0.73	C^{11}, C^{9}, C^{8}
H12	1.01 / 0.73	C^{12}, C^{9}, C^{8}
H14	8.28 / 7.73	H^{14}, C^{14}, C^{13}, N^{e}
NH	6.96 / 9.64	H^{f}, N^{f}, C^{15}, C^{16}
H16	4.39 / 2.90	H^{16}, C^{16}, N^{f}
H17	1.64 / 1.54	H^{17}, C^{17}, C^{16}
H18	1.64 / 1.60	C^{18}, C^{17}, C^{19}
H18’	0.89 / 0.44	C^{18}, C^{17}, C^{19}
H19	0.82 / 1.00	H^{19}, C^{19}, C^{18}
H20	1.64 / 0.80	C^{20}, C^{21}, C^{19}
H20’	0.89 / 0.80	C^{20}, C^{21}, C^{19}
H21	2.12 / 1.78	H^{21}, C^{21}, C^{16}
H22	0.82 / 1.88	C^{22}, C^{21}, C^{23}
H22’	1.58 / 1.88	C^{22}, C^{21}, C^{23}
H23	1.49 / 1.80	H^{23}, C^{23}, C^{22}
H24	2.12 / 1.88	C^{24}, C^{23}, C^{17}
H24’	1.83 / 1.88	C^{24}, C^{23}, C^{17}
H25	0.82 / 1.74	C^{25}, C^{23}, C^{19}
H25’	-0.03 / 1.74	C^{25}, C^{23}, C^{19}

Table S2. Experimental ^{13}C chemical shifts and atoms aligned for AZD8329. The chemical shifts are obtained from Ref. 1.

Label	Experimental shift, Form 1 / Form 4 [ppm]	Atoms aligned
C1	173.60 / 171.04	C^1, O^a, O^b, H^a
C2	133.27 / 131.10	C^2, C^3, H^3, O^b
C3	131.50 / 133.01	C^3, H^3, O^a, O^b
C4	127.00 / 128.05	C^4, H^4, N^d, C^1
C5	148.27 / 147.31	C^5, N^d, C^4, C^6
C6	128.32 / 128.05	C^6, H^6, N^d, C^1
C7	131.50 / 130.48	C^7, H^7, O^a, O^b
C8	151.97 / 148.71	C^8, N^d, C^9, C^{13}
C9	34.20 / 33.42	C^9, C^8, C^{10}
C10	30.13 / 29.53	C^{10}, C^9, C^8
C11	30.13 / 29.53	C^{11}, C^9, C^8
C12	30.13 / 29.53	C^{12}, C^9, C^8
C13	119.17 / 114.10	C^{13}, C^8, C^{14}, C^{15}
C14	139.16 / 138.43	C^{14}, N^e, C^{13}, H^{14}
C15	165.41 / 172.98	C^{15}, N^f, O^c
C16	55.24 / 60.16	C^{16}, N^f, H^{16}
C17	32.13 / 32.45	C^{17}, C^{16}, C^{18}, C^{24}
C18	32.13 / 30.80	C^{18}, C^{17}, C^{19}
C19	27.26 / 27.81	C^{19}, C^{18}, C^{20}, C^{25}
C20	32.13 / 30.80	C^{20}, C^{21}, C^{19}
C21	32.79 / 34.14	C^{21}, C^{16}, C^{20}, C^{22}
C22	37.32 / 37.41	C^{22}, C^{21}, C^{23}
C23	26.93 / 27.81	C^{23}, C^{22}, C^{24}, C^{25}
C24	38.83 / 36.42	C^{24}, C^{23}, C^{17}
C25	37.13 / 37.41	C^{25}, C^{23}, C^{19}

Table S3. Experimental ^{13}C-^{1}H chemical shifts and atoms aligned for AZD8329. The chemical shifts are obtained from Ref. 1.

Label	Experimental shift, Form 1 / Form 4 [ppm]	Atoms aligned
C3-H3	131.50, 8.46 / 133.01, 9.01	C^3, H^3, O^a, O^b
C4-H4	127.00, 7.08 / 128.05, 8.47	C^4, H^4, N^d, C^1
C6-H6	128.32, 8.46 / 128.05, 6.92	C^6, H^6, N^d, C^1
C7-H7	131.50, 8.46 / 130.48, 8.69	C^7, H^7, O^a, O^b
C10-H10	30.13, 1.01 / 29.53, 0.73	C^{10}, C^9, C^8
C11-H11	30.13, 1.01 / 29.53, 0.73	C^{11}, C^9, C^8
C12-H12	30.13, 1.01 / 29.53, 0.73	C^{12}, C^9, C^8
C14-H14	139.16, 8.28 / 138.43, 7.73	C^{14}, N^e, C^{13}, H^{14}
C16-H16	55.24, 4.39 / 60.16, 2.90	C^{16}, N^f, H^{16}
C17-H17	32.13, 1.64 / 32.45, 1.54	C^{17}, C^{16}, C^{18}, C^{24}
C18-H18	32.13, 1.64 / 30.80, 1.60	C^{18}, C^{17}, C^{19}
C18-H18’	32.13, 0.89 / 30.80, 0.44	C^{18}, C^{17}, C^{19}
C19-H19	27.26, 0.82 / 27.81, 1.00	C^{19}, C^{18}, C^{20}, C^{25}
C20-H20	32.13, 1.64 / 30.80, 0.80	C^{20}, C^{21}, C^{19}
C20-H20’	32.13, 0.89 / 30.80, 0.80	C^{20}, C^{21}, C^{19}
C21-H21	32.79, 2.12 / 34.14, 1.78	C^{21}, C^{16}, C^{20}, C^{22}
C22-H22	37.32, 0.82 / 37.41, 1.88	C^{22}, C^{21}, C^{23}
C22-H22’	37.32, 1.58 / 37.41, 1.88	C^{22}, C^{21}, C^{23}
C23-H23	26.93, 1.49 / 27.81, 1.80	C^{23}, C^{22}, C^{24}, C^{25}
C24-H24	38.83, 2.12 / 36.42, 1.88	C^{24}, C^{23}, C^{17}
C24-H24’	38.83, 1.83 / 36.42, 1.88	C^{24}, C^{23}, C^{17}
C25-H25	37.13, 0.82 / 37.41, 1.74	C^{25}, C^{23}, C^{19}
C25-H25’	37.13, -0.03 / 37.41, 1.74	C^{25}, C^{23}, C^{19}

Table S4. Experimental ^{1}H chemical shifts and atoms aligned for decitabine. The chemical shifts are obtained from Ref. 2.

Label	Experimental shift [ppm]	Atoms aligned
N^bH	9.38	N^b, C^2, N^a, N^c
N^bH’	10.81	N^b, C^2, N^a, N^c
H3	8.30	H^3, N^a, N^d
H4	5.66	H^4, C^4, O^f, N^d
H5	1.83	C^5, C^4, C^6
H5’	1.96	C^5, C^4, C^6
H6	4.08	H^6, C^6, O^g
O^gH	5.90	O^gH, O^g, C^6
H7	3.33	H^7, C^7, O^f
H8	3.91	C^8, O^h, C^7
H8’	3.36	C^8, O^h, C^7
O^hH	5.90	O^hH, O^h, C^8

Table S5. Experimental ^{13}C chemical shifts and atoms aligned for decitabine. The chemical shifts are obtained from Ref. 2.

Label	Experimental shift [ppm]	Atoms aligned
C1	153.75	C^1, O^e, N^c, N^d
C2	165.97	C^2, N^a, N^b, N^c
C3	153.75	C^3, N^a, N^d
C4	88.61	C^4, O^f, N^d, C^5
C5	44.97	C^5, C^4, C^6
C6	72.23	C^6, O^g, H^6
C7	98.73	C^7, O^f, H^7
C8	62.12	C^8, O^h, C^7

Table S6. Experimental ^{13}C-^{1}H chemical shifts and atoms aligned for decitabine. The chemical shifts are obtained from Ref. 2.

Label	Experimental shift [ppm]	Atoms aligned
C3-H3	153.75, 8.30	C^3, N^a, N^d
C4-H4	88.61, 5.66	C^4, O^f, N^d, C^5
C5-H5	44.97, 1.83	C^5, C^4, C^6
C5-H5’	44.97, 1.96	C^5, C^4, C^6
C6-H6	72.23, 4.08	C^6, O^g, H^6
C7-H7	98.73, 3.33	C^7, O^f, H^7
C8-H8	62.12, 3.91	C^8, O^h, C^7
C8-H8’	62.12, 3.36	C^8, O^h, C^7

Table S7. Experimental ^{1}H chemical shifts and atoms aligned for lisinopril dihydrate. The chemical shifts are obtained from Ref. 3.

Label	Experimental shift [ppm]	Atoms aligned
H1	7.8	H^1, C^2, C^7
H2	6.3	H^2, C^2, C^7
H3	7.6	H^3, C^3, C^7
H5	7.9	H^5, C^5, C^7
H6	7.6	H^6, C^6, C^7
H7	3.8	C^7, C^4, C^8
H8	2.1	C^8, C^7, C^9
H9	4.6	H^9, C^9, N^f
N^fH	11.3	N^f, C^9, C^{11}
H11	4.5	H^{11}, C^{11}, N^f
H12	1.7	C^{12}, C^{11}, C^{13}
H13	0.7	C^{13}, C^{12}, C^{14}
H14	0.2	C^{14}, C^{13}, C^{15}
H14’	1.5	C^{14}, C^{13}, C^{15}
H15	0.2	C^{15}, C^{14}, N^g
H15’	2.6	C^{15}, C^{14}, N^g
H17	5.2	C^{17}, N^h, C^{18}

H18	1.6	C^{18}, C^{17}, C^{19}
H19	1.6	C^{19}, C^{18}, C^{20}
H20	4.4	H^{20}, C^{20}, N^{h}

Table S8. Experimental ^{13}C chemical shifts and atoms aligned for lisinopril dihydrate. The chemical shifts are obtained from Ref. 3.

Label	Experimental shift [ppm]	Atoms aligned
C1	127.4	C^{1}, C^{2}, C^{7}
C2	128.7	C^{2}, H^{2}, C^{7}
C3	130.1	C^{3}, H^{3}, C^{7}
C4	142.3	C^{4}, C^{3}, H^{3}
C5	128.2	C^{5}, H^{5}, C^{7}
C6	130.1	C^{6}, H^{6}, C^{7}
C7	30.9	C^{7}, C^{4}, C^{8}
C8	35.2	C^{8}, C^{7}, C^{9}
C9	56.4	C^{9}, N^{f}, H^{9}
C10	173.9	C^{10}, O^{a}, O^{b}
C11	54.6	C^{11}, N^{f}, H^{11}
C12	28.3	C^{12}, C^{11}, C^{13}
C13	18.9	C^{13}, C^{12}, C^{14}
C14	27.2	C^{14}, C^{13}, C^{15}
C15	35.9	C^{15}, C^{14}, N^{g}
C16	164.4	C^{16}, O^{c}, N^{h}
C17	47.6	C^{17}, N^{h}, C^{18}
C18	25.3	C^{18}, C^{17}, C^{19}
C19	30.9	C^{19}, C^{18}, C^{20}
C20	61.2	C^{20}, N^{h}, H^{20}
C21	175.7	C^{21}, O^{d}, O^{e}

Table S9. Experimental ^{13}C-^{1}H chemical shifts and atoms aligned for lisinopril dihydrate. The chemical shifts are obtained from Ref. 3.

Label	Experimental shift [ppm]	Atoms aligned
C1-H1	127.4, 7.8	C^{1}, C^{2}, C^{7}
C2-H2	128.7, 6.3	C^{2}, H^{2}, C^{7}
C3-H3	130.1, 7.6	C^{3}, H^{3}, C^{7}
C5-H5	128.2, 7.9	C^{5}, H^{5}, C^{7}
C6-H6	130.1, 7.6	C^{6}, H^{6}, C^{7}
C7-H7	30.9, 3.8	C^{7}, C^{4}, C^{8}
C8-H8	35.2, 2.1	C^{8}, C^{7}, C^{9}
C9-H9	56.4, 4.6	C^{9}, N^{f}, H^{9}
C11-H11	54.6, 4.5	C^{11}, N^{f}, H^{11}
C12-H12	28.3, 1.7	C^{12}, C^{11}, C^{13}
C13-H13	18.9, 0.7	C^{13}, C^{12}, C^{14}
C14-H14	27.2, 0.2	C^{14}, C^{13}, C^{15}
C14-H14’	27.2, 1.5	C^{14}, C^{13}, C^{15}
C15-H15	35.9, 0.2	C^{15}, C^{14}, N^{g}

C15-H15’	35.9, 2.6	C^{15}, C^{14}, N^{g}
C17-H17	47.6, 5.2	C^{17}, N^{h}, C^{18}
C18-H18	25.3, 1.6	C^{18}, C^{17}, C^{19}
C19-H19	30.9, 1.6	C^{19}, C^{18}, C^{20}
C20-H20	61.2, 4.4	C^{20}, N^{h}, H^{20}

Table S10. Experimental ^{1}H chemical shifts and atoms aligned for AZD5718. The chemical shifts are obtained from Ref. 4.

Label	Experimental shift [ppm]	Atoms aligned
H1	1.2	C^{1}, C^{2}, C^{3}, N^{d}
H3	5.8	H^{3}, C^{3}, C^{2}, C^{4}
N^{d}H	10.6	N^{d}H, N^{d}, N^{e}, C^{2}
H6	6.9	H^{6}, C^{6}, C^{4}
H7	6.7	H^{7}, C^{7}, C^{11}
H9	7.0	H^{9}, C^{9}, C^{11}
H10	7.3	H^{10}, C^{10}, C^{4}
H12	3.9	H^{12}, C^{12}, C^{11}
H13	0.0	C^{13}, C^{12}, C^{14}
H13’	1.7	C^{13}, C^{12}, C^{14}
H14	-0.5	C^{14}, C^{13}, C^{15}
H14’	0.8	C^{14}, C^{13}, C^{15}
H15	-0.5	C^{15}, C^{14}, C^{16}
H15’	0.8	C^{15}, C^{14}, C^{16}
H16	1.6	C^{16}, C^{15}, C^{17}
H16’	1.6	C^{16}, C^{15}, C^{17}
H17	1.6	H^{17}, C^{17}, C^{18}
N^{f}H	7.7	N^{f}H, N^{f}, C^{18}, C^{19}
H20	7.6	H^{20}, C^{20}, N^{g}, C^{19}
H22	1.7	C^{22}, N^{h}, C^{23}
H22’	2.7	C^{22}, N^{h}, C^{23}
H23	1.9	C^{23}, N^{i}, C^{22}
H23’	2.7	C^{23}, N^{i}, C^{22}
N^{i}H	6.9	N^{i}H, N^{i}, C^{23}, C^{24}

Table S11. Experimental ^{13}C chemical shifts and atoms aligned for AZD5718. The chemical shifts are obtained from Ref. 4.

Label	Experimental shift [ppm]	Atoms aligned
C1	11.1	C^1, C^2, C^3, N^d
C2	141.5	C^2, C^1, C^3, N^d
C3	102.3	C^3, C^2, C^4
C4	149.8	C^4, C^3, C^5, N^e
C5	139.5	C^5, C^4, C^6, N^e
C6	123.9	C^6, H^6, C^4
C7	130.1	C^7, H^7, C^{11}
C8	133.3	C^8, O^a, C^7
C9	130.8	C^9, H^9, C^{11}
C10	125.3	C^{10}, H^{10}, C^4
C11	201.1	C^{11}, O^a, C^{12}, C^8
C12	46.3	C^{12}, H^{12}, C^{11}
C13	31.2	C^{13}, C^{12}, C^{14}
C14	26.6	C^{14}, C^{13}, C^{15}
C15	26.0	C^{15}, C^{14}, C^{16}
C16	29.2	C^{16}, C^{15}, C^{17}
C17	49.8	C^{17}, H^{17}, C^{16}
C18	174.0	C^{18}, O^b, N^f
C19	125.8	C^{19}, N^f, C^{20}, C^{21}
C20	130.8	C^{20}, H^{20}, N^g, C^{19}
C21	119.7	C^{21}, N^h, C^{19}, C^{24}
C22	43.5	C^{22}, N^h, C^{23}
C23	40.1	C^{23}, N^i, C^{22}
C24	161.8	C^{24}, C^{21}, O^c, N^i

Table S12. Experimental ^{13}C-^{1}H chemical shifts and atoms aligned for AZD5718. The chemical shifts are obtained from Ref. 4.

Label	Experimental shift [ppm]	Atoms aligned
C1-H1	11.1, 1.2	C^1, C^2, C^3, N^d
C3-H3	102.3, 5.8	C^3, C^2, C^4
C6-H6	123.9, 6.9	C^6, H^6, C^4
C7-H7	130.1, 6.7	C^7, H^7, C^{11}
C9-H9	130.8, 7.0	C^9, H^9, C^{11}
C10-H10	125.3, 7.3	C^{10}, H^{10}, C^4
C12-H12	46.3, 3.9	C^{12}, H^{12}, C^{13}
C13-H13	31.2, 0.0	C^{13}, C^{12}, C^{14}
C13-H13’	31.2, 1.7	C^{13}, C^{12}, C^{14}
C14-H14	26.6, -0.5	C^{14}, C^{13}, C^{15}
C14-H14’	26.6, 0.8	C^{14}, C^{13}, C^{15}
C15-H15	26.0, -0.5	C^{15}, C^{14}, C^{16}
C15-H15’	26.0, 0.8	C^{15}, C^{14}, C^{16}
C16-H16	29.2, 1.6	C^{16}, C^{15}, C^{17}
C16-H16’	29.2, 1.6	C^{16}, C^{15}, C^{17}
C17-H17	49.8, 1.6	C^{17}, H^{17}, C^{16}
C20-H20	130.8, 7.6	C^{20}, H^{20}, N^g, C^{19}
C22-H22	43.5, 1.7	C^{22}, N^h, C^{23}
C22-H22’	43.5, 2.7	C^{22}, N^h, C^{23}
C23-H23	40.1, 1.9	C^{23}, N^i, C^{22}
C23-H23’	40.1, 2.7	C^{23}, N^i, C^{22}

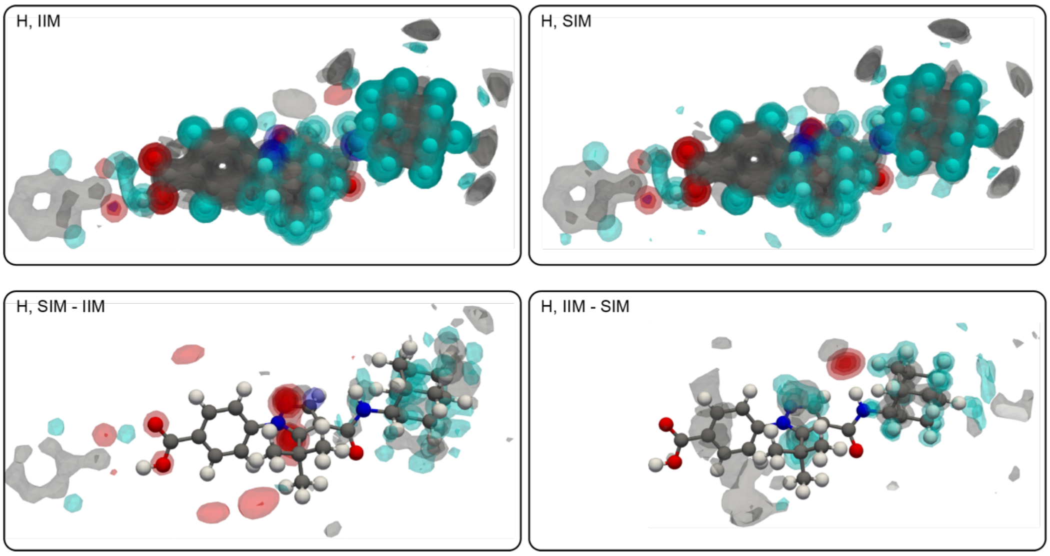

Figure S2. Interaction maps of AZD8329 Form 1 based on ^{1}H chemical shifts.

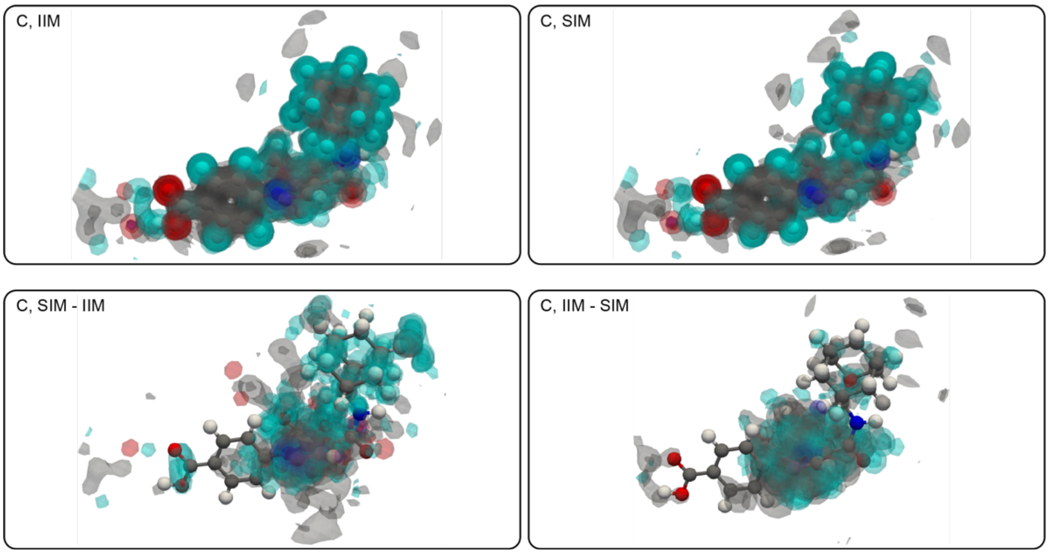

Figure S3. Interaction maps of AZD8329 Form 1 based on ^{13}C chemical shifts.

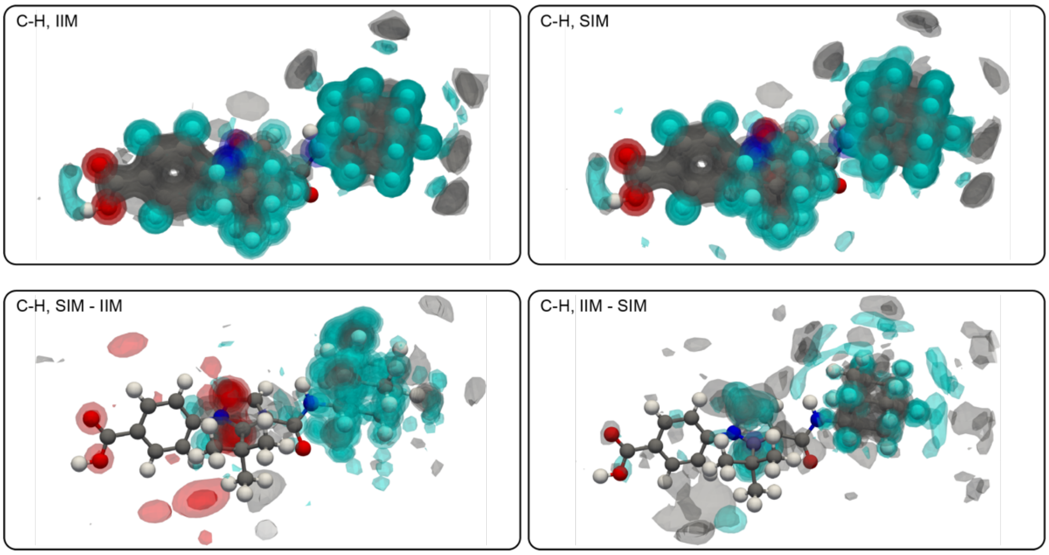

Figure S4. Interaction maps of AZD8329 Form 1 based on ^{1}H and ^{13}C chemical shifts.

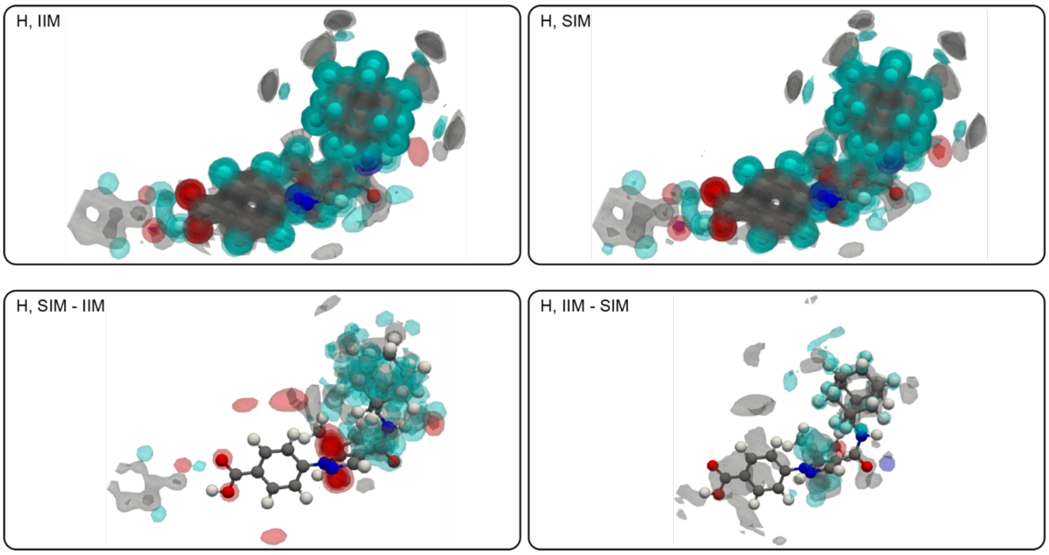

Figure S5. Interaction maps of AZD8329 Form 4 based on ^{1}H chemical shifts.

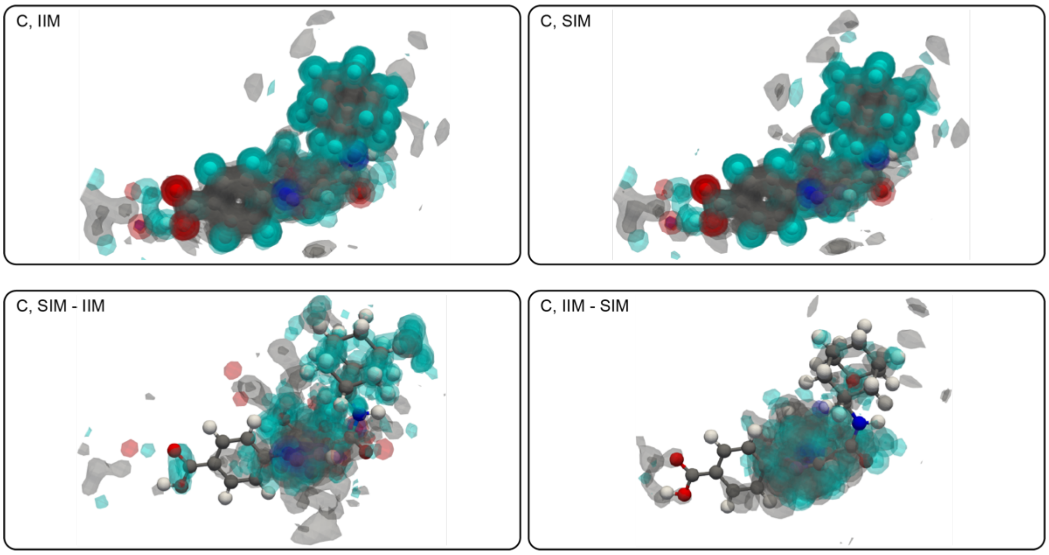

Figure S6. Interaction maps of AZD8329 Form 4 based on ^{13}C chemical shifts.

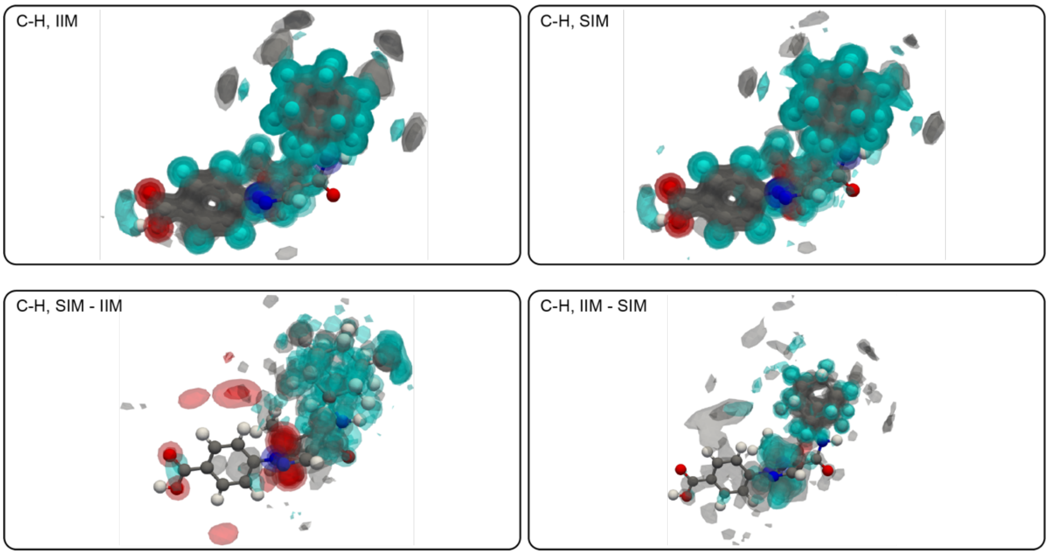

Figure S7. Interaction maps of AZD8329 Form 4 based on ^{1}H and ^{13}C chemical shifts.

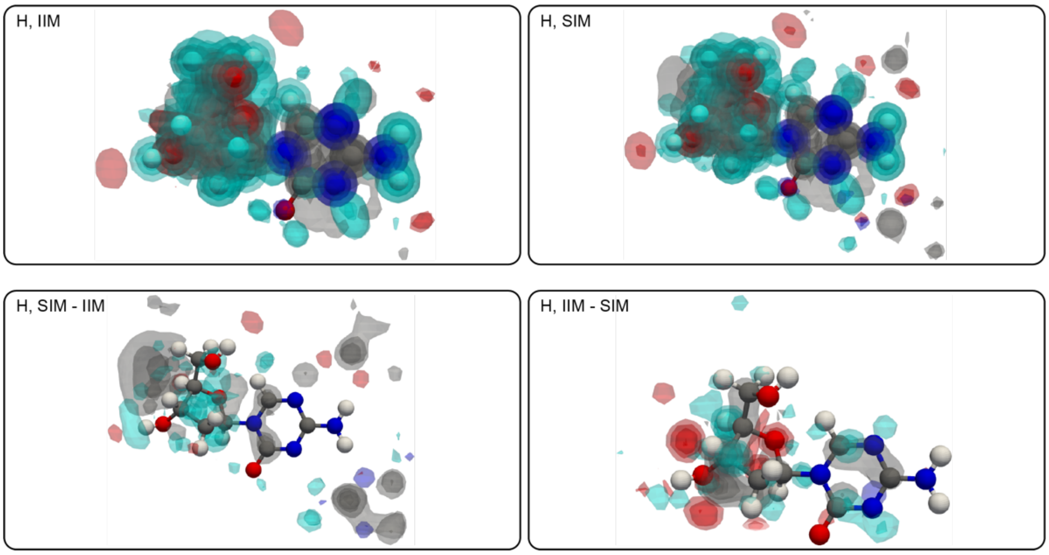

Figure S8. Interaction maps of decitabine based on ^{1}H chemical shifts.

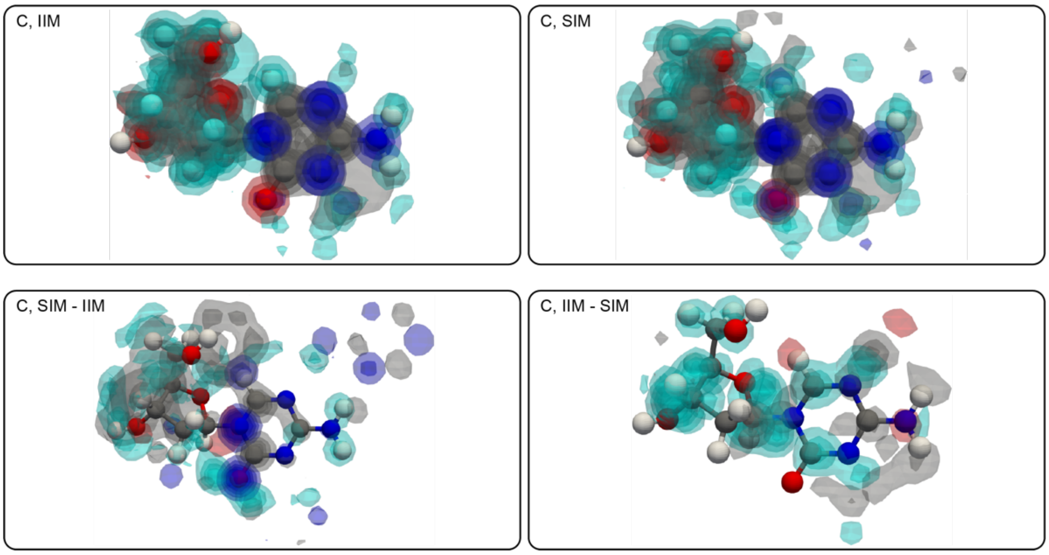

Figure S9. Interaction maps of decitabine based on ^{13}C chemical shifts.

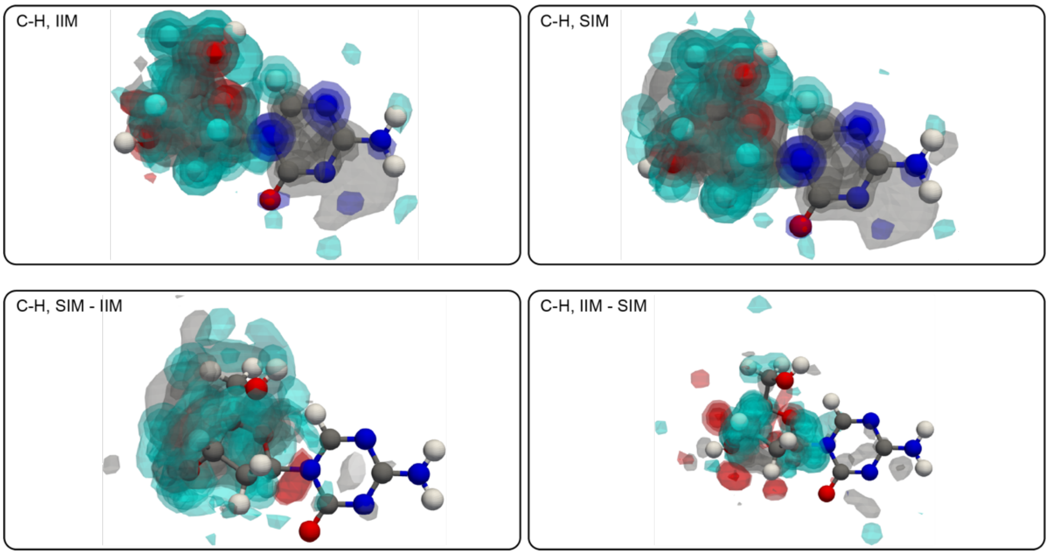

Figure S10. Interaction maps of decitabine based on ^{1}H and ^{13}C chemical shifts.

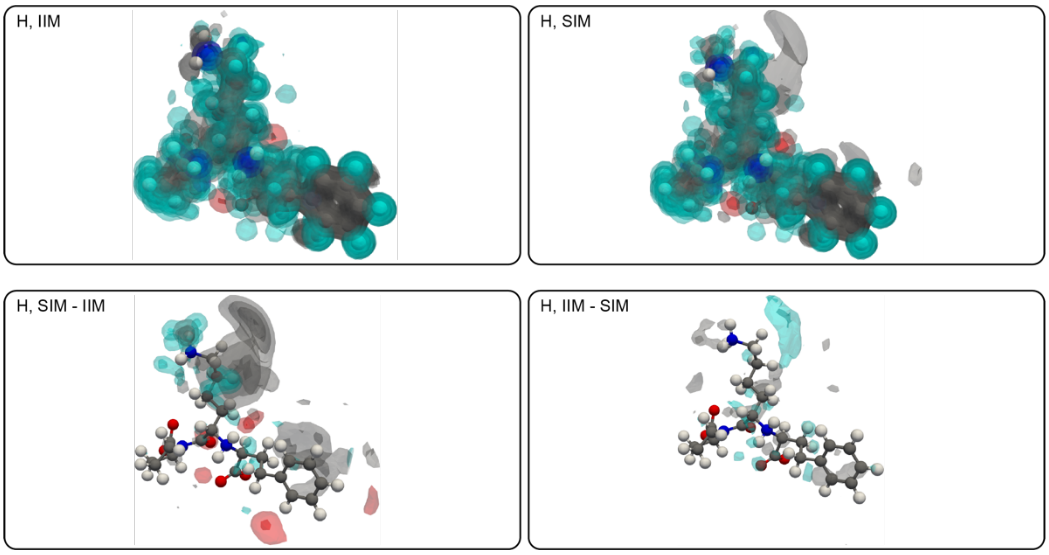

Figure S11. Interaction maps of lisinopril dihydrate based on ^{1}H chemical shifts.

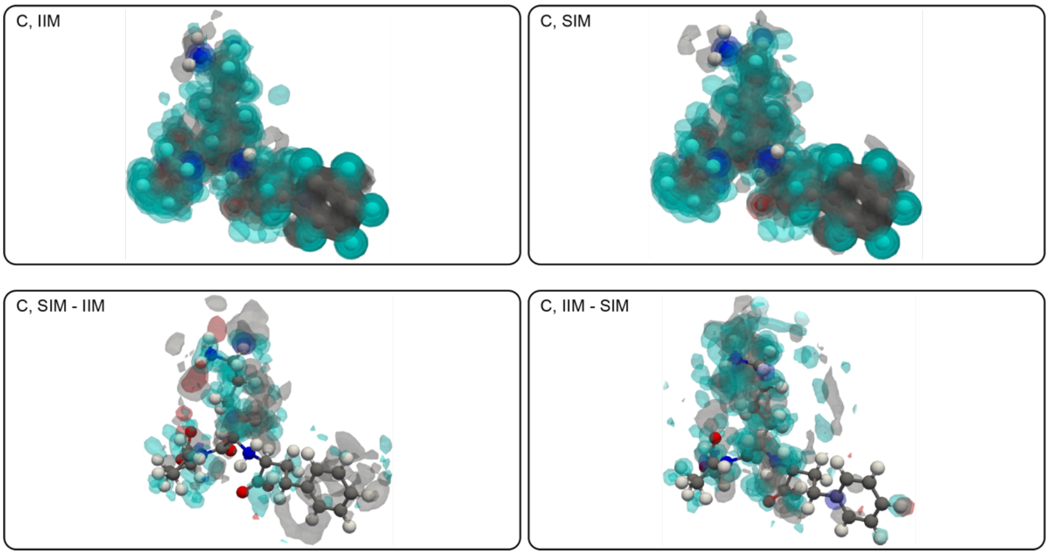

Figure S12. Interaction maps of lisinopril dihydrate based on ^{13}C chemical shifts.

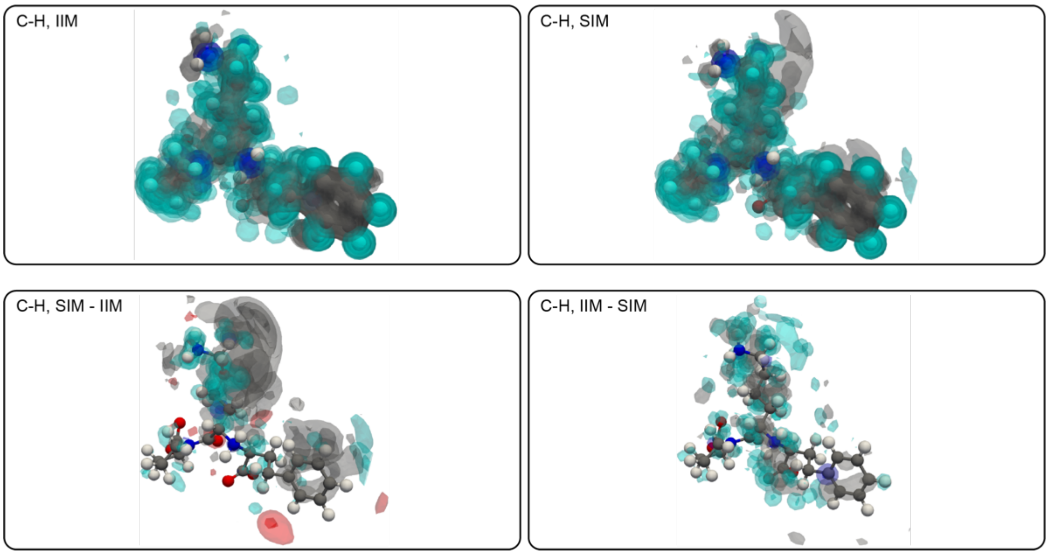

Figure S13. Interaction maps of lisinopril dihydrate based on ^{1}H and ^{13}C chemical shifts.

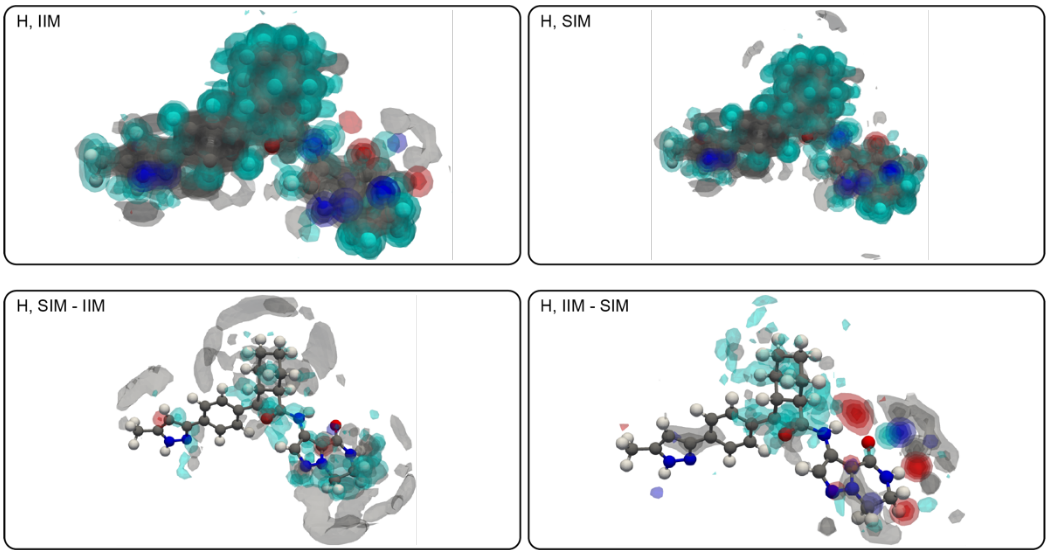

Figure S14. Interaction maps of AZD5718 based on ^{1}H chemical shifts.

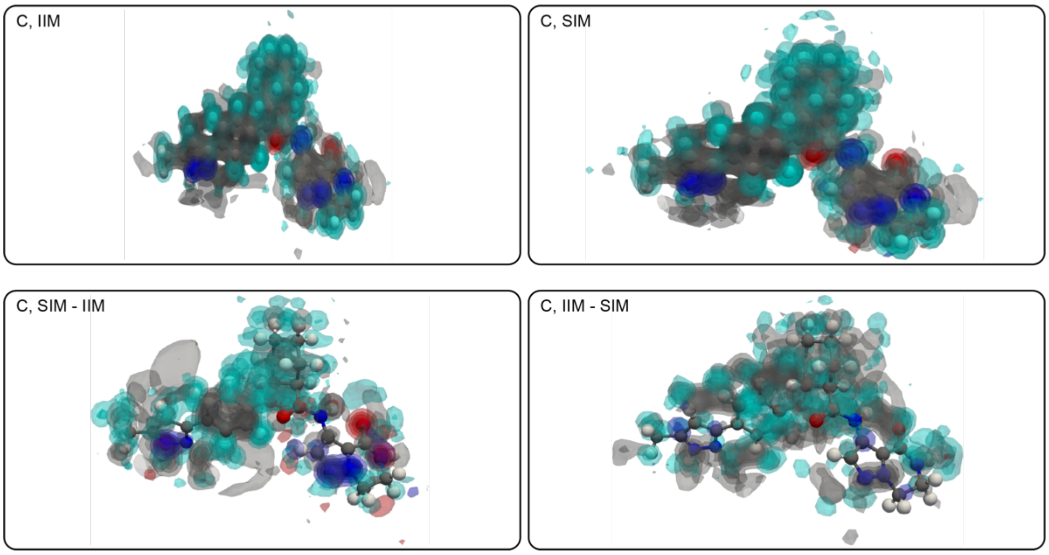

Figure S15. Interaction maps of AZD5718 based on ^{13}C chemical shifts.

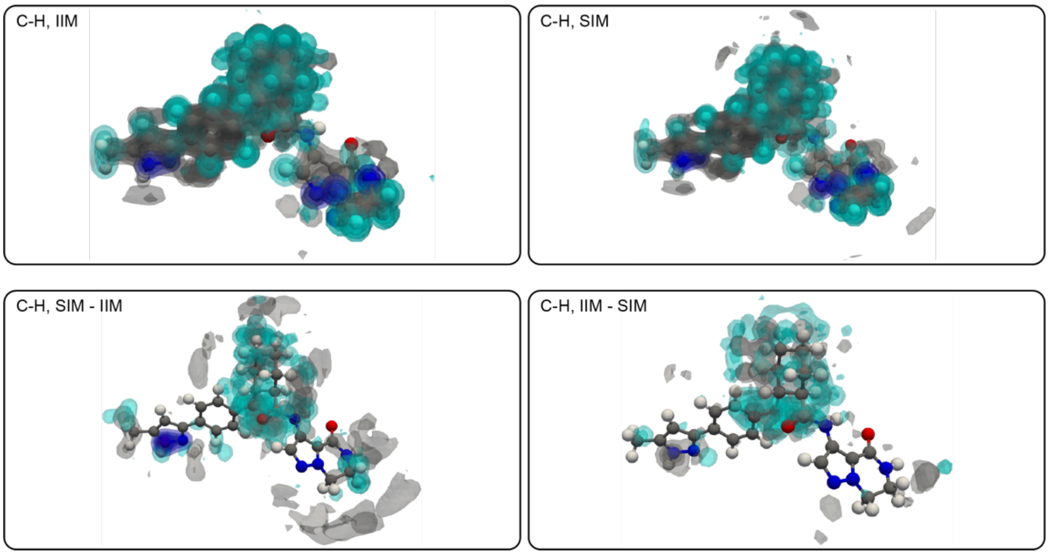

Figure S16. Interaction maps of AZD5718 based on ^{1}H and ^{13}C chemical shifts.

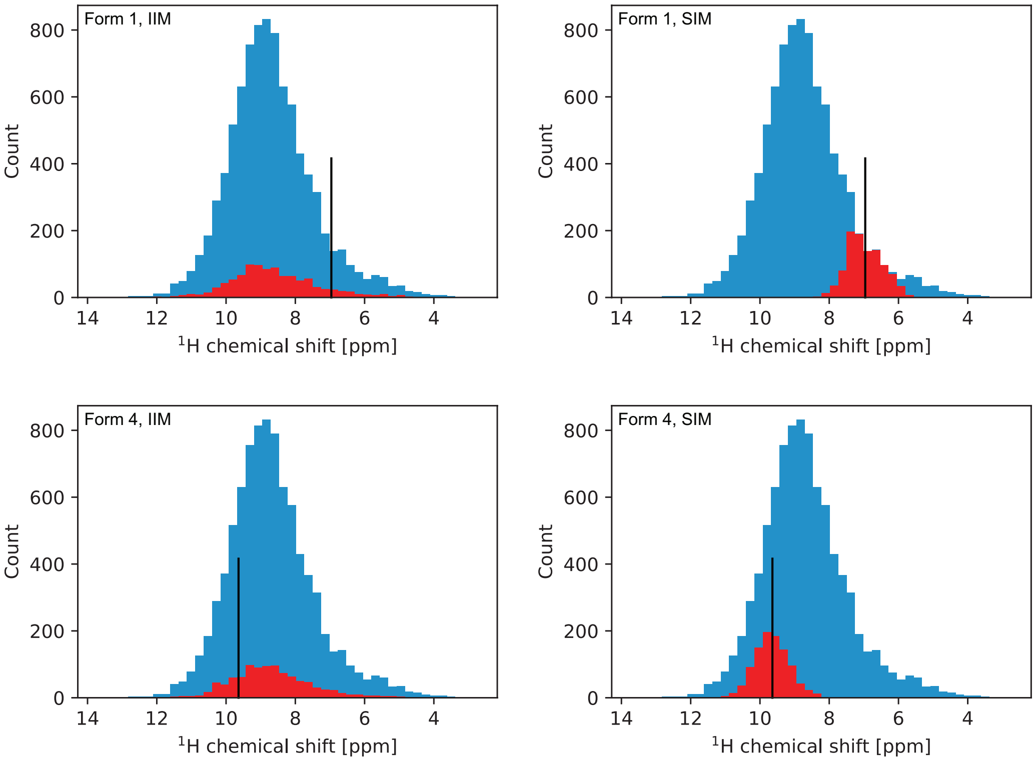

Figure S17. Histogram of ^{1}H chemical shifts from the database matching the local covalent environment of the NH proton (blue) and selected environments (red) used to construct the IIM (left) and SIM (right) for AZD8329 form 1 (top) and form 4 (bottom). The experimental shifts are indicated by the vertical black lines.

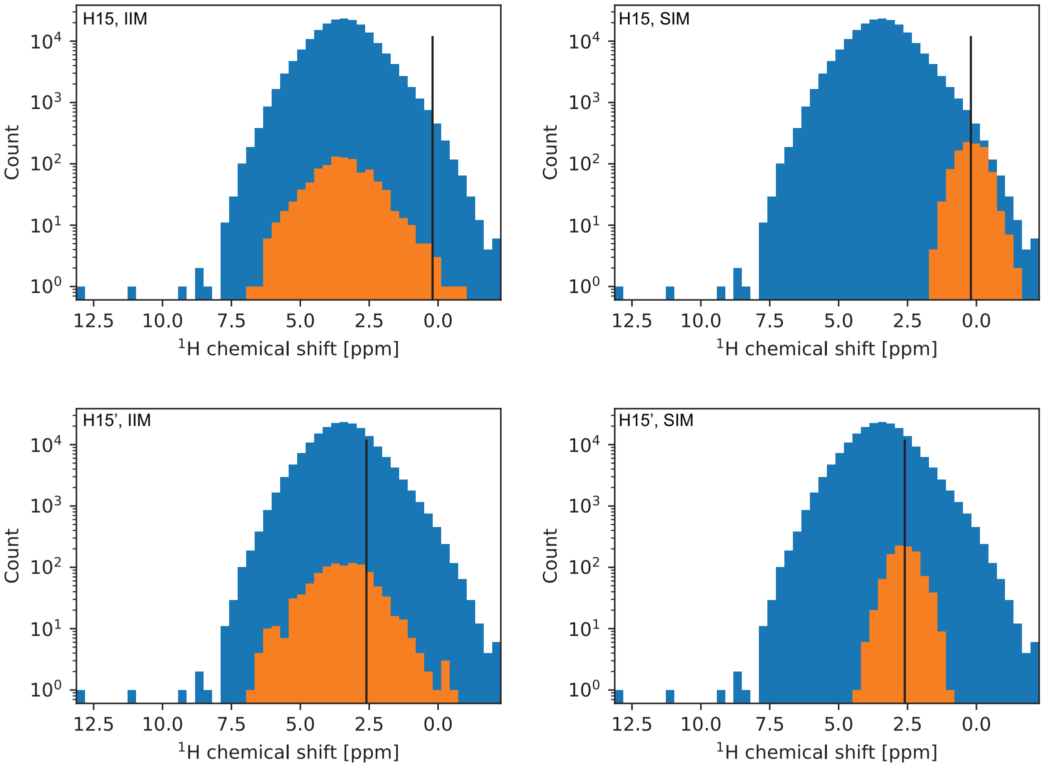

Figure S18. Histogram of ^{1}H chemical shifts from the database matching the local covalent environment of proton labelled **15** (blue) and selected environments (orange) used to construct the IIM (left) and SIM (right). The experimental shift is indicated by the vertical black line.

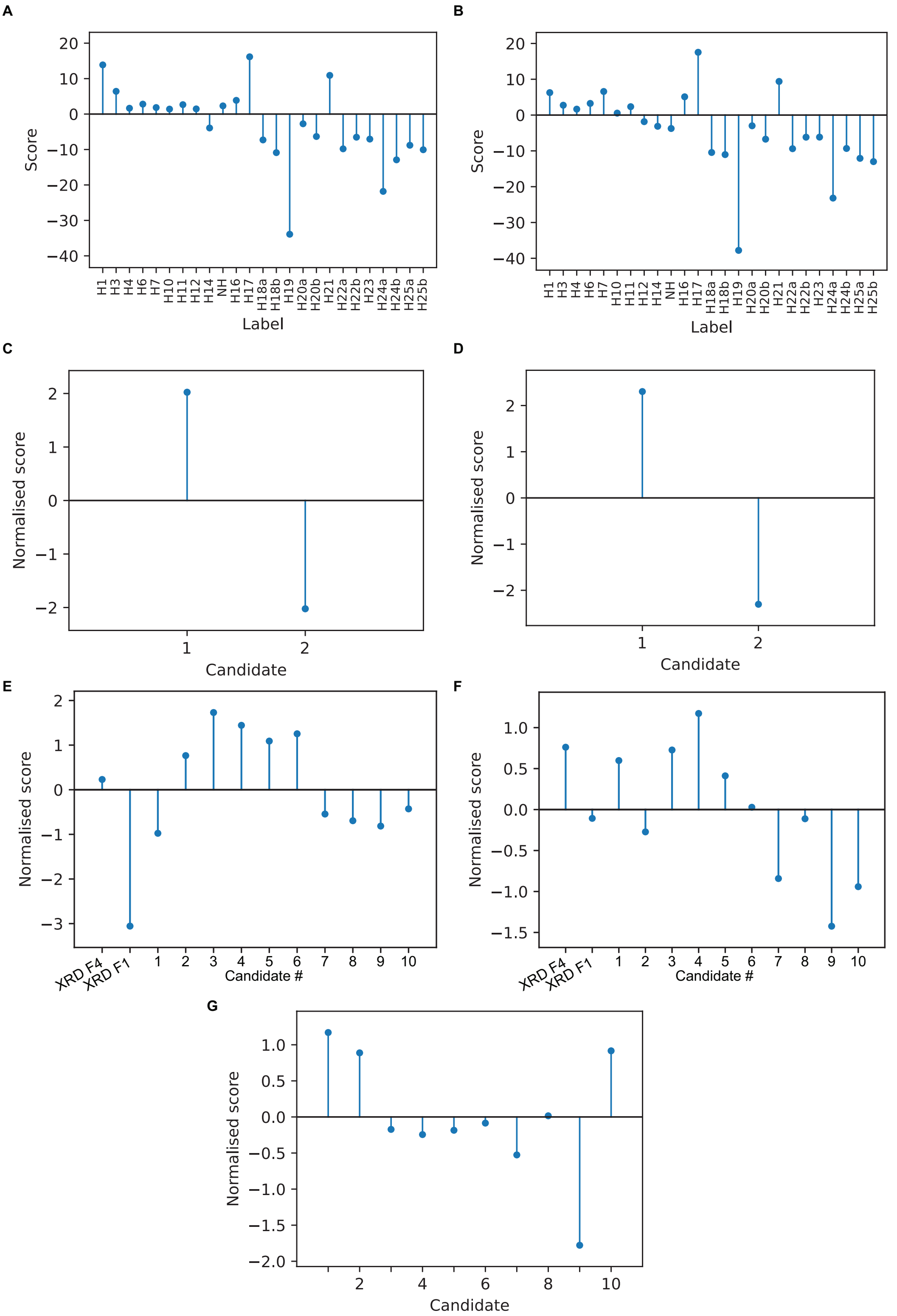

Figure S19. (**A**), (**B**) Scores of individual atoms of the X-ray structures of AZD8329 forms 1 and 4, respectively, using SIMs constructed using the experimentally obtained chemical shifts of AZD8329 form 1. (**C**), (**D**) Scores of the X-ray structures of AZD8329 forms 1 (candidate 1) and 4 (candidate 2) using experimental ^{13}C and ^{1}H-^{13}C chemical shifts of AZD8329 form 1, respectively. The scores obtained using ^{1}H-^{13}C chemical shifts were obtained after discarding shifts from the adamantane group in the molecule. (**E**), (**F**) Scores of the X-ray structures of AZD8329 forms 1 and 4 and of the CSP set for AZD8329 form 4 using experimental ^{13}C and ^{1}H-^{13}C chemical shifts of AZD8329 form 4, respectively. The scores obtained using ^{1}H-^{13}C chemical shifts were obtained after discarding shifts from the adamantane group in the molecule. (**G**) Scores of the CSP set of AZD5718 using experimental ^{13}C chemical shifts.

References

1. Baias, M.; Dumez, J.-N.; Svensson, P. H.; Schantz, S.; Day, G. M.; Emsley, L., De Novo Determination of the Crystal Structure of a Large Drug Molecule by Crystal Structure Prediction-Based Powder NMR Crystallography. *J Am Chem Soc* **2013,** *135* (46), 17501-17507.

2. Brus, J.; Czernek, J.; Kobera, L.; Urbanova, M.; Abbrent, S.; Husak, M., Predicting the Crystal Structure of Decitabine by Powder NMR Crystallography: Influence of Long-Range Molecular Packing Symmetry on NMR Parameters. *Crystal Growth & Design* **2016,** *16* (12), 7102-7111.

3. Miclaus, M.; Grosu, I.-G.; Filip, X.; Tripon, C.; Filip, C., Optimizing structure determination from powders of crystalline organic solids with high molecular flexibility: the case of lisinopril dihydrate. *CrystEngComm* **2014,** *16* (3), 299-303.

4. Cordova, M.; Balodis, M.; Hofstetter, A.; Paruzzo, F.; Nilsson Lill, S. O.; Eriksson, E. S. E.; Berruyer, P.; Simões de Almeida, B.; Quayle, M. J.; Norberg, S. T.; Svensk Ankarberg, A.; Schantz, S.; Emsley, L., Structure determination of an amorphous drug through large-scale NMR predictions. *Nat Commun* **2021,** *12*, 2964.
